# Photocatalytic Diselenide Contraction as a Tool for Site‐Selective Isosteric Ubiquitylation

**DOI:** 10.1002/psc.70037

**Published:** 2025-07-03

**Authors:** Herwig Weissinger, Moritz Urschbach, Luca Ferrari, Sascha Martens, Christian F. W. Becker

**Affiliations:** ^1^ Faculty of Chemistry, Institute of Biological Chemistry University of Vienna Vienna Austria; ^2^ Max Perutz Labs Vienna Biocenter Campus (VBC) Vienna Austria; ^3^ Max Perutz Labs University of Vienna Vienna Austria

## Abstract

Ubiquitylation is a highly conserved post‐translational modification (PTM) in eukaryotes, which serves as a critical regulatory mechanism for protein homeostasis, cellular transport, signal transduction pathways, and numerous other functions. The biological function of ubiquitylation is dictated predominantly by the topology of its linkage. Deciphering ubiquitin's complex biochemistry necessitates novel synthetic methods that deliver well‐defined, biosimilar ubiquitylation. To this end, a semisynthetic strategy relying on the recombinant expression of ubiquitin combined with chemoselective photocatalytic diselenide contraction (PDC) was established to enable site‐selective biomimetic selenalysine‐linked ubiquitylation. The modification of ubiquitin with a C‐terminal selenol was fine‐tuned to avoid hydrolysis. The conditions of the PDC reaction, such as solvent composition, phosphine concentration, and irradiation, were optimized for efficient ubiquitylation of a Tau F derived peptide. Furthermore, it was demonstrated that the selenalysine linkage undergoes efficient cleavage by deubiquitylating enzymes, comparable to the native isopeptide linkage. The presented method expands the toolbox of site‐selective ubiquitylation techniques. It is tolerant to many functional groups and will help to further elucidate the complexities of ubiquitylation.

## Graphical abstract

Chemoselective photocatalytic diselenide contraction (PDC) enables site‐selective biomimetic ubiquitylation of substrate peptides. The resulting selenalysine linkage undergoes efficient cleavage by deubiquitylating enzymes.

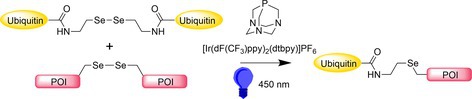

## 1 Introduction

Ubiquitylation is one of the central post‐transitional modifications (PTMs) by which regulation of cellular processes in eukaryotes is achieved. This modification involves covalent attachment of ubiquitin, a small protein of 76 amino acids which remains mostly conserved since the last common ancestor of all eukaryotes [1]. Early research identified (poly‐)ubiquitin as a tag for proteasomal degradation [2]. Ubiquitylation is now recognized as a structurally diverse PTM, with impact on many cellular processes such as signaling, cell cycle control, and cellular localization.

Typically, ubiquitylation of proteins proceeds via formation of an isopeptide linkage between the C‐terminus of ubiquitin and the *ε*‐amino group of lysine in the target protein. However, ubiquitylation of other nucleophilic groups in proteins as well as in other biomolecules such as lipids and RNA has been documented [3]. Ubiquitin itself features eight amino groups amenable to ubiquitylation (seven lysine side chains as well as the N‐terminus) giving rise to a vast number of possible polyubiquitin assemblies. Such assemblies comprise linear and branched chains as well as homotypic and heterotypic varieties, the former consisting of one type of isopeptide linkage and the latter of multiple types. Depending on the linkage topology, polyubiquitylation can elicit diverse biological effects, which is referred to as the ubiquitin code [4].

To precisely install ubiquitylations on a large variety of target proteins, cells employ a cascade of three enzymes (E1–E3). The E1 enzymes activate the C‐terminus of ubiquitin as a thioester. Consequently, the ubiquitin is transferred to an E2 enzyme by transthioesterification [5]. In this reaction cascade, most E3 enzymes only act as mediators of selectivity by binding both the E2 enzyme as well as the substrate protein. The fact that E3 enzymes mediate selectivity is reflected by the large number of different E3 ligases, accounting for about 5% of the human genome [6].

**SCHEME 1:**
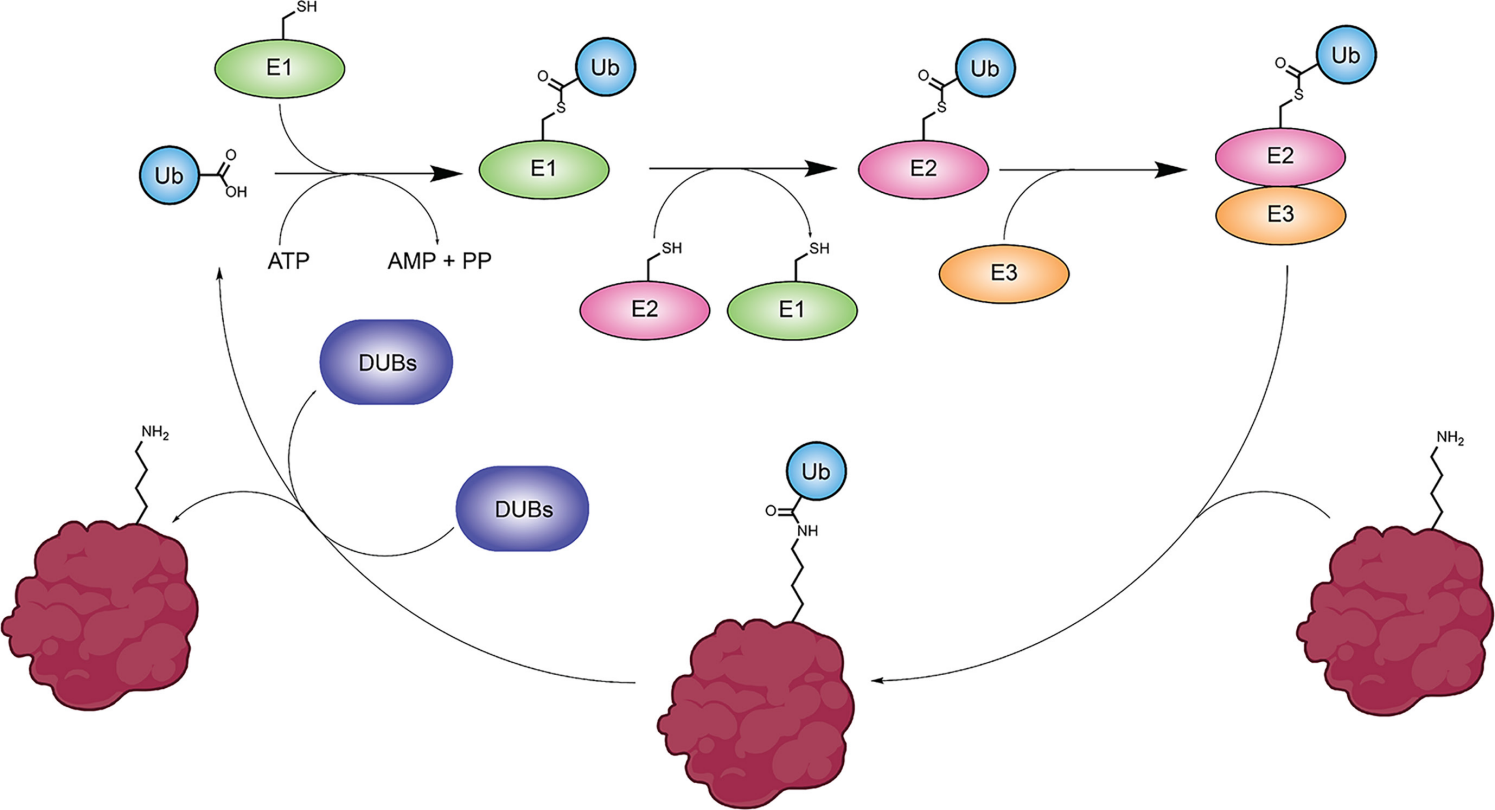
Schematic overview of ubiquitylation through E1, E2, E3 enzymes and deubiquitylation mediated by DUBs.

To ensure fast‐paced and dynamic control of cellular processes, ubiquitylation must be a reversible process. Deubiquitylating enzymes (DUBs) function antagonistically to the ubiquitylation cascade by removing or editing ubiquitin tags (Scheme 1) [7].

## 2 Synthetic Methods for Ubiquitylation

**SCHEME 2:**
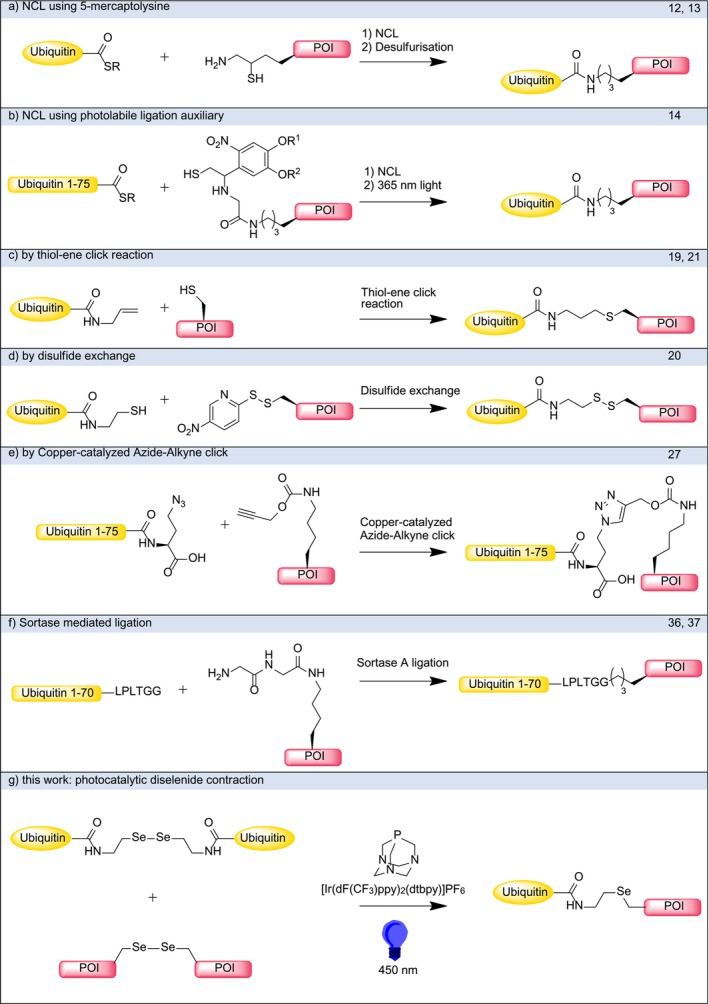
(a–g) Overview of (semi‐)synthetic routes toward ubiquitylated proteins of interest (POI).

In order to elucidate the biological outcome of protein ubiquitylation, methods that deliver well defined and regioselective ubiquitylation of peptides and proteins are needed. *In cellulo*, ubiquitylation generally does not lead to homogenously ubiquitylated proteins, but instead produces complex mixtures of ubiquitylated proteins that cannot be separated. While enzymatic ubiquitylations based on the E1, E2, and E3 cascade can be utilized in vitro, the choice and availability of E2 and E3 enzymes to target specific proteins of interest remains challenging [8]. This strategy is further complicated by the promiscuity of some ubiquitin conjugating enzymes, which leads to undesired isomers [9]. If a specific polyubiquitylation is needed, the product is generally of low chain homogeneity (ranging from mono‐ to polyubiquitylated species). This necessitates complex purification procedures such as cation exchange and size exclusion chromatography or the use of protected ubiquitin precursors [10]. To avoid these complications, a variety of synthetic approaches have been developed to generate ubiquitin chains and to attach ubiquitin to proteins of interest (POIs) (Scheme 2).

Arguably the best method to obtain a native isopeptide linked ubiquitylation is native chemical ligation (NCL) [11], yielding products which are structurally indistinguishable from the native products. Therefore, these compounds are perfectly suited for biochemical and biophysical studies. Scheme 2 summarized two NCL‐based methods, one uses mercaptolysine [12, 13], whereas the other approach is based on a photolabile ligation auxiliary (Scheme 2a,b) [14]. Many other auxiliaries such as 2‐(aminooxy)ethanethiol, which can be removed by reduction, have been described in the literature [15]. To enable NCL reactions, another strategy employs genetic code expansion for the covalent incorporation of d‐Cys‐*ε*‐Lys residues into POIs [16]. However, the utility of NCL‐based methods is greatly hampered by the fact that the POI must contain non‐proteinogenic amino acids. This remains difficult for proteins outside the size range of solid phase peptide synthesis, requiring additional engineering, such as genetic code expansion. A closely related method using a thiazolidine protected 5‐mercaptolysine building block has been used by the Brik group to assemble tetraubiquitin in a stepwise manner [17].

Another possibility for site specific ubiquitylation and synthesis of polyubiquitin chains is via the use of orthogonal lysine protecting groups. Boc‐*ε*‐Lys is introduced at the desired site via genetic code expansion, followed by global Cbz‐protection of the other amines. Boc‐deprotection and subsequent silver‐mediated amide formation leads to native linkages and allows complete regioselectivity [18].

Besides constructing native isopeptide bonds by NCL, other chemoselective reactions have been employed to deliver a linkage one atom longer than the natural isopeptide linkage (Scheme 2c) [19, 20, 21]. For the thialysine linkage created by the thiol‐ene click reaction, it has been demonstrated that it is sufficiently similar in geometry and reactivity to be used as a surrogate for in vitro studies [22]. While the disulfide linkage is reasonably easy to generate and close to the native linkage, it is sensitive to reducing conditions and it remains unclear if there are biological differences such as recognition by deubiquitylating enzymes (Scheme 2d). Many other methods targeting thiol groups in POIs exist, such as thiol‐maleimide chemistry [23] and electrophilic functionalization using chloroacetaldehyde, which can subsequently be used to establish an oxime linkage [24]. This extends the scope of chemical ubiquitylation to proteins of any size, provided they only possess one accessible thiol moiety. When the lack of the native isopeptide linkage is acceptable, these methods are generally preferred due to their facile nature and superior yield.

Genetic code expansion allows for installation of bioorthogonal functional groups such as in azidohomoalanine and propargyl‐derivatized lysine, which can be used in a copper‐catalyzed azide‐alkyne click reaction to form the 1,4‐disubstituted triazole linkage (Scheme 2e). An analogous triazole linkage has been obtained by the use of *p*‐azidophenylalanine with a propargylamide‐terminated ubiquitin [25]. In an alternative approach, a photocaged aminooxy‐l‐lysine has been introduced via genetic code expansion and used to form a nonhydrolyzable oxime linkage [26]. Although similar in size and structure, the created linkage is not hydrolyzed by DUBs or other degrading enzymes, a fact that is exploited to identify new binding partners of ubiquitylated proteins via affinity purification [27]. The proteolytic stability of non‐native ubiquitylation presents a successful strategy to study DUB binding [28], substrate recognition of DUBs [29] and has found utility as DUB inhibitors [30]. Furthermore, such linkages provide opportunities to discover interactions of ubiquitylated proteins and their downstream processing [31, 32]. This approach offers high versatility in terms of protein substrates and has been used to assemble diubiquitin [33].

Sortase A, a transamidase enzyme found in *Staphylococcus aureus* , recognizes the LPXTG sequence in proteins which it cleaves between threonine and glycine to allow attack of a substrate with one or more N‐terminal glycine residues [34] (Scheme 2f). To create the desired isopeptide linkage, *ε*‐diglycyl lysine is incorporated into the POI. This has been achieved by incorporation of the corresponding azide protected amino acid using genetic code expansion and subsequent Staudinger reduction [35]. Although this method furnishes a native isopeptide linkage and works under physiological conditions, as a consequence of the mutations at C‐terminus, this ubiquitylation is not cleaved by DUBs. The main advantages are the mild conditions (possible in vitro as well as *in cellulo*) and the avoidance of denaturing conditions [36]. This strategy enables the construction of diverse polyubiquitin chain topologies, including tetraubiquitin and branched ubiquitin conjugates [37].

We were inspired by the work of Dowman et al. carried out in collaboration with us [38], in which we demonstrated photocatalyzed diselenide contraction (PDC) as a tool providing access to peptide‐small molecule conjugates, peptide dimers, and C‐terminally modified ubiquitin derivatives carrying a C‐terminal selenol. The reaction was initially described by Waliczek et al., who irradiated diselenides at 254 nm for 1 h without any photocatalyst or phosphine to achieve such a contraction reaction [39]. Recently, the group of Payne described an electrochemical approach to achieve this transformation, further improving both yield and selectivity [40].

We hypothesized that this methodology could be used for site selective ubiquitylation of peptides. The PDC strategy utilizes the unique reactivity of selenocysteine which can be introduced through solid phase peptide synthesis (SPPS), stop codon suppression [41], or the auxotrophy method [42]. The resulting selenalysine linkage is isosteric to the native isopeptide linkage and therefore potentially useful in the study of ubiquitin's complex biochemistry.

## 3 Results and Discussion

**SCHEME 3:**
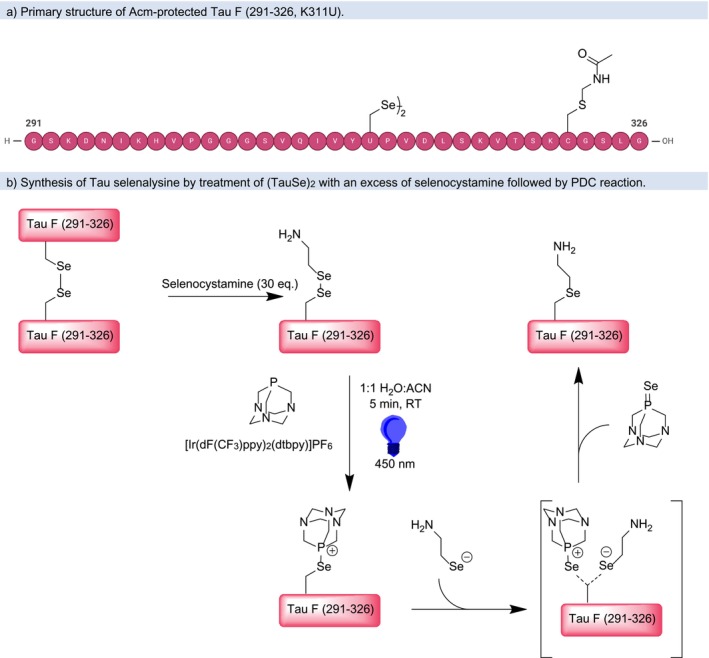
(a) Primary structure of Acm‐protected Tau F (291‐326, K311U) after Mob‐deprotection. (b) Synthesis of Tau selenalysine by treatment of (TauSe)_2_ with an excess of selenocystamine followed by PDC reaction.

In order to establish the PDC reaction for ubiquitylation, we chose a peptide segment corresponding to a repeat domain of Tau F (aa 291–326). Incorporation of Sec at Position 311 replacing a Lys (K311U mutation) provides a peptide that can undergo a PDC reaction at this frequently ubiquitylated amino acid, which is a PTM often observed in Alzheimer's disease patients [43]. SPPS delivered the orthogonally protected Cys side chain (protected with acetamidomethyl, Acm) and Sec side chain (4‐methoxybenzyl, Mob) in good yield (Scheme 3a). The Acm group was needed to eliminate participation of the cysteine thiol in the PDC chemistry, however a selective removal route for Mob was sought. Here, a two‐step procedure, first converting the methoxybenzyl protecting group to 5‐nitropyridyl selenylsulfide using bis(5‐nitro‐2‐pyridyl) disulfide (DTNP) in TFA, followed by reduction using dithiothreitol (DTT) in water, was most effective. The desired methoxybenzyl‐deprotected peptide ((TauSe)_2_) was produced in near quantitative yield (25.9 mg) and high purity (Supporting Information S2.1).

In order to test this reaction with (TauSe)_2_, the heterodiselenide was formed by incubation with an excess of selenocystamine. Since the outcome of diselenide scrambling is stochastic, a large excess of one of the homodiselenides is used to ensure complete conversion of the other. The reaction requires a highly concentrated solution of at least 0.5 mM for the limiting reagent and consequently the excess reagent needs to have higher solubility, which restricts the choice of possible substrates. After equilibration and formation of the mixed diselenide, the photocatalyst [Ir(dF(CF_3_)ppy)_2_(dtbpy)]PF_6_ was added as a solution in ACN and 1,3,5‐triaza‐7‐phosphaadamantane (PTA) is added as a freshly prepared solution in water (see Supporting Information S1.6).

The mechanism of the PDC reaction, as described by the group of Payne, consists of heterolytic scission of the diselenide, leading to a selenate and a selanylphosphonium compound (Scheme 3b). In an S_N_2 process, the selenate attacks the Sec‐β‐carbon, displacing the phosphine selenide and leading to the selenoether product [38]. A similar reaction has been demonstrated for disulfides, which give the contraction reaction in the presence of phosphines and without a photocatalyst [44, 45].

Upon addition of the catalyst, the phosphine and irradiation for 5 min incomplete conversion of the starting material was observed. Further addition of phosphine and repeated irradiation resulted in complete consumption of the starting material. The presence of the selenoether product was confirmed using LC–MS, and a yield of ~60% (HPLC) was calculated (Supporting Information S2.2).

**SCHEME 4:**
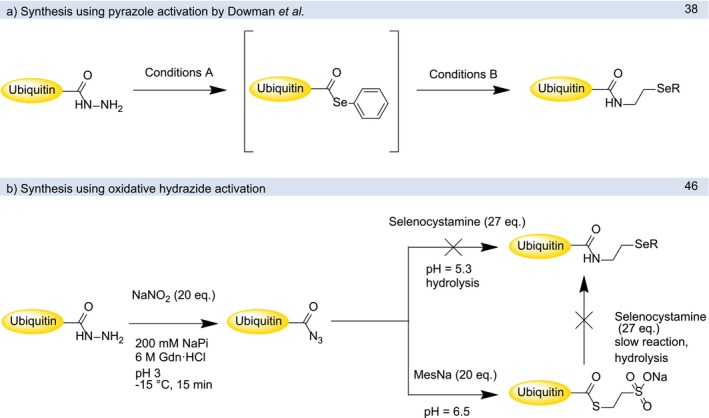
Synthesis of (UbNHCH_2_CH_2_Se)_2_: (a) via phenyl selenoester (Conditions A and B are described in Table 1) and (b) via acyl azide.

Upon successfully establishing the PDC reaction with (TauSe)_2_, we focused on the generation of C‐terminally selenated ubiquitin. The expression of a Ub‐Mxe‐GyrA‐H7‐CBD fusion construct (ubiquitin is fused here to a cleavable *Mycobacterium xenopi* GyrA intein (Mxe‐GyrA) and a polyhistidine (H7) and Chitin Binding Domain (CBD) for purification [21]) in *Escherichia coli* proceeded smoothly, the desired protein was purified via affinity chromatography using chitin‐beads and finally cleaved from the beads via hydrazinolysis, analogous to previously published conditions [21]. The previously published conditions for the synthesis of (UbNHCH_2_CH_2_Se)_2_ required further optimization to provide enough material for our ubiquitylation reactions (Table 1). We initially tested two alternative conditions using the azide intermediate [46] but these proved unsuccessful (Supporting Information S2.3.2). The acyl azide hydrolyzed too quickly and did not undergo attack by selenocystamine (Scheme 4b). Alternatively, the intermediate MesNa thioester proved too unreactive for substitution with selenocystamine (Scheme 4b). However, we succeeded in optimizing reaction conditions as formation of the phenyl selenoester was more efficient at lower pH, shorter reaction time and increased amounts of acetylacetone (acac). At pH 1.55 and 10 equivalents of acac the reaction was finished in less than 45 min (Table 1). As the product UbNHCH_2_CH_2_SeH hydrolyzes rather quickly at pH 5 under reducing conditions (Supporting Information S2.3.3), the duration and temperature of the second step were reduced to 10 min at −21 °C. To accelerate the reaction, 50 equivalents of selenocystamine were used. Phenylselenol and DPDS were removed by extraction using Et_2_O. The extraction step proved essential to prevent the undesired formation of UbNHCH_2_CH_2_SeSePh, which would give rise to undesired Ub‐phenyl selenoethers during the PDC reaction. To prevent hydrolysis during extraction, the aqueous phase was frozen in dry ice/acetone which allowed for the Et_2_O layer to be decanted and the aqueous phase was thawed at −21°C. The product was purified via preparative RP‐HPLC to yield the corresponding Ub‐selenocystamide, which oxidized to the corresponding diselenide during purification/storage. These improved conditions led to negligible hydrolysis and a total yield of 91% (59.4 mg) (Supporting Information S2.3.1).

**TABLE 1:** Optimization of conditions for synthesis of (UbNHCH_2_CH_2_Se)_2_.

Conditions A^a^	Conditions B	Results
5 eq. Acac, 2.5 h, pH 2.5	5 eq. Selenocystamine, pH 5, 0.5 h, RT	< 50% yield
10 eq. Acac, 2 h, pH 1.6	5 eq. Selenocystamine, pH 5, 0.5 h, RT	~50% yield
10 eq. Acac, 1 h, pH 1.56	50 eq. Selenocystamine, pH 5, 0.5 h, RT	45% yield
10 eq. Acac, 1 h, pH 1.52	50 eq. Selenocystamine, pH 5, 0.5 h, 0°C	61% yield
10 eq. Acac, 1 h, pH 1.56	50 eq. Selenocystamine, pH 5, 10 min, −20°C	82% yield^b^
10 eq. Acac, 45 min, pH 1.55	50 eq. Selenocystamine, pH 5, 10 min, −20°C, Et_2_O extraction	91% yield

^a^ 200 mM TCEP, 50 mM DPDS, 6 M Gdn·HCl, 200 mM HEPES at RT.

^b^ 69% of (UbNHCH_2_CH_2_Se)_2_ 25% of UbNHCH_2_CH_2_SeSePh, 82% in total.

**FIGURE 1:**
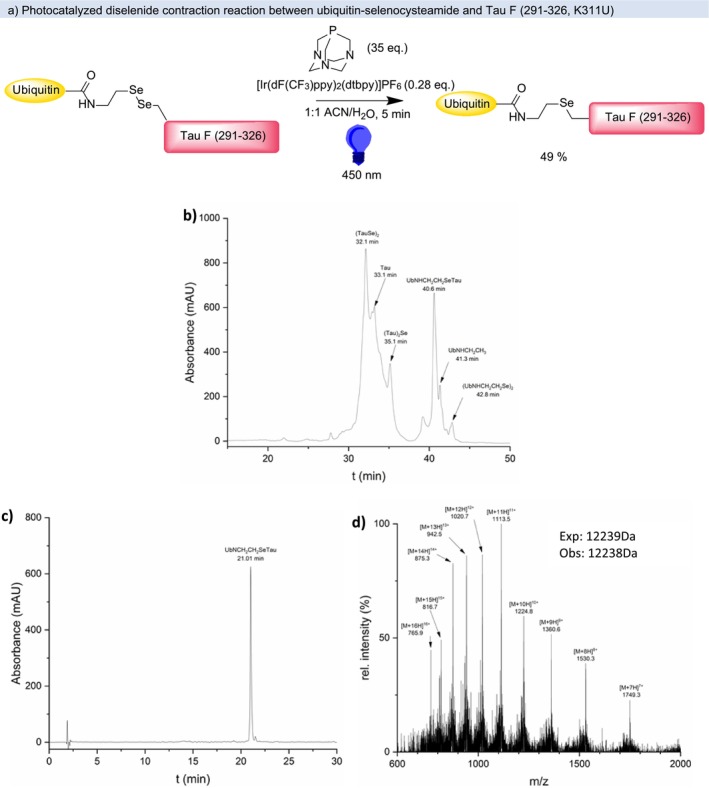
(a) Photocatalyzed diselenide contraction reaction between ubiquitin‐selenocystamide and Tau F (291–326, K311U), characterization of UbNHCH_2_CH_2_SeTau; (b) preparative RP‐HPLC chromatogram of the crude (Gradient C, 60°C, PerfectSil 300 C4 10 μm, 250 × 10 mm, 214 nm); (c) RP‐HPLC chromatogram at 214 nm, (d) ESI MS+.

Having successfully synthesized both the precursor (TauSe)_2_ and the ubiquitin selenocystamide precursor (UbNHCH_2_CH_2_Se)_2_, we proceeded to test the PDC reaction using these substrates. While Dowman et al. used a large excess of the small molecule diselenide (> 32 eq.), to guarantee complete formation of the heterodiselenide, this was not practical with peptide substrates due to their restricted solubility and availability. Instead, eight equivalents of (TauSe)_2_ were used, which should (assuming stochastic exchange) lead to 89% heterodiselenide. Dowman et al. found four equivalents PTA (with respect to the diselenides) to be ideal, but when applied to our substrates, eight equivalents of PTA were required to give complete conversion of the starting material (Supporting Information S2.4.3). It was found that switching the solvent to a 1:1 mixture of ACN:H_2_O without any buffer or chaotropic agents improved peptide solubility slightly so this mixture was used in all further experiments unless stated otherwise. Employing these improved conditions, the desired TauF‐Ub selenoether was isolated in 49% (1.0 mg) yield and high purity (Figure 1 and Supporting Information S2.4.4).

Unfortunately, the need for an excess of one of the diselenides severely limits the use of the PDC reaction for more complex peptides and protein substrates. Furthermore, the excess homodiselenide is deselenized over the course of the PDC reaction and cannot be recovered for subsequent reactions. In an attempt to avoid these issues, we investigated pre‐formed and purified heterodiselenides as substrates. Different approaches for the targeted synthesis of heterodiselenides were evaluated [47]. Although the reaction of UbNHCH_2_CH_2_SeH with Tau 5‐nitropyridyl selenylsulfide [20] produced the heterodiselenide in an acceptable yield (~50% by HPLC), the stochastic exchange method was employed due to its simplicity (Supporting Information S2.5.1). It was hypothesized that a photochemically generated selanyl radical could also react with Tau 5‐nitropyridyl selenylsulfide to produce the desired heterodiselenide [48]. The experiment demonstrated the successful formation of the target heterodiselenide as the most prominent product; however, side products arising from prolonged irradiation at 360 nm diminished its synthetic utility (Supporting Information S2.5.2). The ubiquitin‐Tau heterodiselenide was obtained in 37% yield by equilibrating both homodiselenides and purification via HPLC (Supporting Information S2.5.3).

When purified heterodiselenides were exposed to PDC reaction conditions, they quickly equilibrated back to a mixture of homodiselenides and heterodiselenides [49] faster than the PDC reaction could occur, as evidenced by the formation of (UbNHCH_2_CH_2_Se)_2_ and (TauSe)_2_. No significant amounts of selenoether product were observed (Supporting Information S2.4.2).

Inspired by previous reports of disulfide contraction driven by either UV‐irradiation or aminophosphine reagents [50, 51, 52] and the fact that the mechanism proposed by Dowman et al. could conceivably be extended to selenylsulfides as well, we wanted to investigate whether selenylsulfides would undergo a similar contraction reaction. This would extend the scope of the reaction to cysteine containing peptides and proteins. Initial attempts to synthesize selenylsulfides via exchange of disulfides and diselenides did not produce the product in appreciable quantities (Supporting Information S2.6.1). By using the 5‐nitropyridyl selenylsulfide as an activated selenium species and the corresponding thiol as the nucleophile, selenylsulfides could be synthesized in good yield. When irradiated under PDC conditions, selenylsulfides gave diselenides, thiols, and thiaphosphine products, but no desired thio‐ or selenoether products (Supporting Information S2.6.2).

**FIGURE 2:**
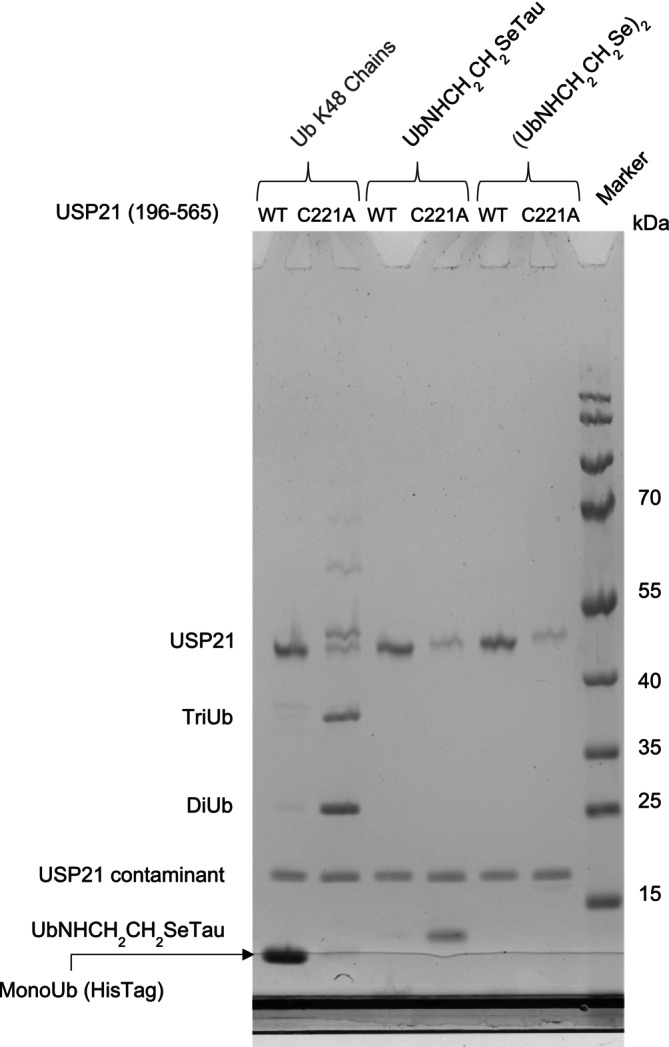
DUB essay: Odd lanes contain the wild type USP21 (196–565), even lanes contain the inactive mutant USP21 (196–565, C221A). The first two lanes (from left to right) were charged with polyubiquitin chains, the following two with selenalysine linked ubiquitylated Tau and the last two with ubiquitin selenocystamine. The very last lane is used for a protein size marker.

In order to demonstrate the biological relevance of selenalysine linked ubiquitylation, the Tau‐ubiquitin selenoether was evaluated as a substrate for deubiquitylating enzymes. Tau‐ubiquitin selenoether was incubated with active USP21 (196–565) and with its inactive mutant USP21 (196–565, C221A). As a positive control for enzyme activity we used substrate‐free K48 connected polyubiquitin chains, which were broken down to monoubiquitin by active USP21 but not by the inactive mutant. Here we could demonstrate that selenalysine‐linked ubiquitylation is also recognized and degraded by USP21, as proven by disappearance of the product band, whereas this band remains in presence of inactive USP21 (Figure 2). The cleaved monoubiquitin is not visible on the NuPage 4%–12% gradient gel shown here; however, on 15% acrylamide gel the cleaved monoubiquitin can be clearly identified (Supporting Information S2.6.3). Based on this analysis, selenalysine‐linked ubiquitylation is a functional isostere for native ubiquitylation and can be applied to generate native‐like ubiquitylation patterns for biological studies.

## 4 Conclusion

The primary aim of this project was to investigate if the PDC reaction can become a viable route to access site‐selective ubiquitylated peptides and proteins similar to other chemoselective reactions such as thiol‐ene chemistry (TEC) [18].

To accomplish this, C‐terminally selenated ubiquitin was prepared from a recombinantly expressed acyl hydrazide precursor. It was discovered that this product hydrolyzes easily under reducing conditions. However, we were able to optimize the synthesis to average yields of > 90%. The intended substrate for ubiquitylation, a selenocysteine containing segment of TauF was prepared via SPPS. Reaction conditions were further optimized using a model reaction between (TauSe)_2_ and selenocystamine yielding selenalysine modified tau peptide in good yields. When applying the PDC reaction to the ubiquitylation of the TauF peptide, we found that this reaction is highly dependent on the PTA concentration and that an increase leads to higher conversion but also deselenization. Using optimized conditions with 8 eq. of PTA yielded 49% ubiquitylated Tau with a selenalysine linkage after isolation, establishing the PDC reaction as a direct method for peptide ubiquitylation.

However, the need for high reactant concentrations and a large excess of Tau peptide are limiting the scope of this reaction. To this end, we aimed at improving reaction efficiency by using preformed heterodiselenides, which would eliminate the need for a large excess of one homodiselenide, hence circumventing the solubility issue associated with a large excess of peptide diselenides. Unexpectedly, the generated heterodiselenides equilibrate to mixtures of homodiselenides and heterodiselenides under PDC reaction conditions and therefore lead to low yields.

Due to the new nature of the selenalysine‐linked ubiquitylation, ubiquitylated Tau peptide was investigated as a substrate for the DUB USP21 to understand if the methylene to selenium exchange affects DUB activity. Ubiquitylated Tau peptide was recognized and cleaved by the deubiquitylating enzyme USP21 at similar rates as enzyme‐derived native polyubiquitin chains.

Overall, we found the PDC reaction to be suitable for peptide monoubiquitylation, however, the required high concentrations limit applicability, as many peptide and protein substrates are not soluble at the required level. This bottleneck could be overcome by applying alternative solvent mixtures or the use of flow‐based chemistry.

## Conflicts of Interest

The authors declare no conflicts of interest.

## Supporting information


**Figure S1** Picture of the irradiation Setup during use with the foil removed for visibility.
**Table S1:** Screening of conditions for selective deprotection of 5‐nitropyridyl.
**Figure S2:** Characterization of Tau F (291–326, K311U): a) Analytical RP‐HPLC chromatogram 214 nm, b) ESI MS, c) Deconvoluted ESI MS (Selenol: Exp: 3649 Da, Obs: 3649 Da; Diselenide: Exp: 7296 Da, Obs: 7295 Da).
**Figure S3:** Characterization of crude Tau (291–326) selenalysine: a) Analytical RP‐HPLC chromatogram at 214 nm, b) LCMS TIC chromatogram, c) ESI MS of peak at 8.5 min, d) Deconvoluted ESI MS of peak at 8.5 min (Exp: 3692 Da, Obs: 3692 Da).
**Figure S4:** Gel electrophoresis a) during expression b) and during purification.
**Figure S5:** Characterization of UbNHNH2: a) Analytical RP‐HPLC chromatogram 214 nm, b) ESI MS, c) Deconvoluted ESI MS (Exp: 8579 Da, Obs: 8577 Da).
**Figure S6:** Characterization of UbNHCH2CH2SeH: a) Analytical RP‐HPLC chromatogram 214 nm, b) ESI MS, c) Deconvoluted ESI MS (Selenol: Exp: 8671 Da, Obs: 8673 Da; Diselenide: Exp: 17340 Da, Obs: 17347 Da).
**Figure S7:** Analysis of the reaction mixture of UbN3 and Selenocystamine: a) ESI MS, b) Deconvoluted ESI MS (UbCOOH: Exp: 8565 Da, Obs: 8563 Da; UbNHCH2CH2SeSeCH2CH2NH2: Exp: 8793 Da, Obs: 8793 Da).
**Figure S8:** Analysis of UbSMeSNa: a) ESI MS, b) Deconvoluted ESI MS (UbCOOH: Exp: 8565 Da, Obs: 8565 Da; UbSMesNa: Exp: 8689 Da, Obs: 8689 Da); Analysis of the reaction mixture of UbSMeSNa and Selenocytsamine after three days: c) ESI MS, d) Deconvoluted ESI MS (UbCOOH: Exp: 8565 Da, Obs: 8565 Da; UbSMesNa: Exp: 8689 Da, Obs: 8689 Da; UbNHCH2CH2SeSeCH2CH2NH2: Exp: 8793 Da, Obs: 8793 Da).
**Figure S9:** Stability of (UbNHCH2CH2Se)2: a) In reducing medium, pH 5, RT (analytical RP‐HPLC, 214 nm) b) In nonreducing medium, pH 5, RT (analytical RP‐HPLC, 214 nm), c) during storage of the lyophilized material at—20 °C (analytical RP‐HPLC, 214 nm, normalized for ease of comparison).
**Scheme S1:** Proposed mechanism for hydrolysis of (UbNHCH2CH2Se)2 at pH 5 under reducing conditions.
**Figure S10:** Analysis of reaction mixture of the PDC reaction between (UbNHCH2CH2Se)2 and (TauSe)2: a) LCMS at 214 nm, b) ESI MS of peak at 9.2 min, c) Deconvoluted ESI MS of peak at 9.2 min (UbCOOH + [O]: Exp: 3692 Da, Obs: 3692 Da; UbNHCH2CH2SeH + 3[O]: Exp: 8719 Da, Obs: 8717 Da; UbNHCH2CH2SeSeTau + [O]: Exp: 12334 Da, Obs: 12332 Da; (UbNHCH2CH2Se)2 + 2[O]: Exp: 17372 Da, Obs: 17371 Da).
**Figure S11:** Analysis of reaction mixture of the PDC reaction between (UbNHCH2CH2Se)2 and the (TauSe)2 under strict exclusion of oxygen: a) LCMS at 214 nm, b) ESI MS of peak at 9.4 min, c) Deconvoluted ESI MS of peak at 9.4 min (UbNHCH2CH3: Exp: 8592 Da, Obs: 8590 Da; UbNHCH2CH2SeH: Exp: 8671 Da, Obs: 8669 Da; UbNHCH2CH2SeTau: Exp: 12239 Da, Obs: 12238 Da; UbNHCH2CH2SeSeTau: Exp: 12318 Da, Obs: 12315 Da; (UbNHCH2CH2Se)2: Exp: 17340 Da, Obs: 17338 Da).
Figure S12: Comparison between 4 equation (1a‐c) and 8 equation (2a‐c) of PTA compared to the total diselenide content for the PDC reaction of (UbNHCH2CH2Se)2 and (TauSe)2: a) LCMS at 214 nm, b) ESI MS of peak at 9.3 min, c) Deconvoluted ESI MS of peak at 9.3 min ((1c): UbNHCH2CH3: Exp: 8592 Da, Obs: 8591 Da; UbNHCH2CH2SeTau: Exp: 12239 Da, Obs: 12238 Da; UbNHCH2CH2SeSeTau: Exp: 12318 Da, Obs: 12316 Da; (UbNHCH2CH2Se)2: Exp: 17340 Da, Obs: 17336 Da, (2c): UbNHCH2CH3: Exp: 8592 Da, Obs: 8591 Da; UbNHCH2CH2SeH: Exp: 8671 Da, Obs: 8669 Da; UbNHCH2CH2SeTau: Exp: 12239 Da, Obs: 12238 Da; (UbNHCH2CH2Se)2: Exp: 17340 Da, Obs: 17337 Da).
**Figure S13:** Analysis of reaction mixture: a) LCMS TIC chromatogram, b) ESI MS of peak at 9.3 min, c) Deconvoluted ESI MS of peak at 9.3 min (UbNHCH2CH3: Exp: 8592 Da, Obs: 8591 Da; UbNHCH2CH2SeTau: Exp: 12239 Da, Obs: 12238 Da).
**Figure S14:** Characterization of UbNHCH2CH2SeTau: a) Preparative chromatogram (Gradient C, 60 °C, PerfectSil 300 C4 10 μm, 250x10 mm, 214 nm), b) RP‐HPLC at 214 nm, c) ESI MS, d) Deconvoluted ESI MS (UbNHCH2CH2SeTau: Exp: 12239 Da, Obs: 12238 Da).
**Table S2:** Screening of removable reducing agents for the reduction of (UbNHCH2CH2Se)2 to yield the corresponding selenol.
**Figure S15:** RP‐HPLC chromatogram of the crude reaction mixture of UbNHCH2CH2SeH and Tau 5‐nitropyridyl selenylsulfide at 214 nm.
**Figure S16:** Analysis of the reaction mixture between (UbNHCH2CH2Se)2 and TauSeSnitropyridyl after irradiation at 360 nm: a) LCMS TIC chromatogram at 3, 10,20 min, b) RP‐HPLC chromatogram after 20 min of irradiation at 214 nm.
**Figure S17:** Characterization of UbNHCH2CH2SeSeTau: a) Analytical RP‐HPLC chromatogram at 214 nm, b) ESI MS, c) Deconvoluted ESI MS (Exp: 12318 Da, Obs: 12316 Da).
**Figure S18:** Characterization of an air equilibrated diselenide‐thiol exchange reaction using UbK48C and (TauSe)2 after 8 d.: a) LCMS at 214 nm, b) ESI MS of peak at 9.5–9‐6 min, c) Deconvoluted ESI MS (Ub(K48C): Exp: 8554 Da, Obs: 8554 Da; Ub(K48C)SSeTau: Exp: 12201 Da, Obs: 12200 Da).
**Figure S19:** Air equilibrated diselenide‐thiol exchange reaction using UbK48C and (TauSe)2 mixture after irradiation: a) LCMS at 214 nm, b) ESI MS of peak at 9.6 min, c) Deconvoluted ESI MS of peak at 9.6
**Figure S20:** Reaction mixture of Tau 5‐nitropyridyl selenylsulfide and ubiquitin K48C after 15 min: a) LCMS TIC chromatogram, b) ESI MS of peak at 9.9 min, c) Deconvoluted ESI MS of peak at 9.9 min (Ub(K48C)SSeTau: Exp: 12201 Da, Obs: 12205 Da).
**Figure S21:** Reaction mixture of Tau 5‐nitropyridyl selenylsulfide and ubiquitin K48C after irradiation: a) LCMS TIC chromatogram, b) ESI MS of peak at 6.9 min, c) Deconvoluted ESI MS of peak at 6.9 min (Ub(K48C): Exp: 12201 Da, Obs: 12205 Da; Ub(K48C) PTA: Exp: 8711 Da, Obs: 8711 Da).
**Figure S22:** DUB essay: Odd lanes contain the wild type USP21 (196–565), even lanes contain the inactive mutant USP21 (196–565, C221A). The first two lanes (from left to right) were charged with polyubiquitin chains, the following two with selenalysine linked ubiquitylated Tau and the last two with ubiquitin selenocystamine. The very last lane is used for a protein size marker.
**Figure S23:** DUB essay using 15% acrylamide gel: Lanes one and four contain the wild type USP21 (196–565), lane three was left empty intentional, lanes two and five contain the inactive mutant USP21 (196–565, C221A). The first two lanes (from left to right) were charged with selenalysine linked ubiquitin, lanes four and five were charged with polyubiquitin chains. The contaminant in lanes one and two, directly above USP21 contaminant band, is likely the homocoupling product (UbNHCH2CH2)2Se.

## Data Availability

The data supporting the findings made herein are made available in the supplementary information or by request to the authors.
