# Supplementary material for: Photocatalytic Diselenide Contraction as a Tool for Site‐Selective Isosteric Ubiquitylation
Source: J Pept Sci. 2025 Jul 3;31(8):e70037. doi: 10.1002/psc.70037 (PMC12226148; doi:10.1002/psc.70037)
Supplement: Supplementary file 1 — Figure S1 Picture of the irradiation Setup during use with the foil removed for visibility. Table S1: Screening of conditions for selective deprotection of 5‐nitropyridyl. Figure S2: Characterization of Tau F (291–326, K311U): a) Analytical RP‐HPLC chromatogram 214 nm, b) ESI MS, c) Deconvoluted ESI MS (Selenol: Exp: 3649 Da, Obs: 3649 Da; Diselenide: Exp: 7296 Da, Obs: 7295 Da). Figure S3: Characterization of crude Tau (291–326) selenalysine: a) Analytical RP‐HPLC chromatogram at 214 nm, b) LCMS TIC chromatogram, c) ESI MS of peak at 8.5 min, d) Deconvoluted ESI MS of peak at 8.5 min (Exp: 3692 Da, Obs: 3692 Da). Figure S4: Gel electrophoresis a) during expression b) and during purification. Figure S5: Characterization of UbNHNH2: a) Analytical RP‐HPLC chromatogram 214 nm, b) ESI MS, c) Deconvoluted ESI MS (Exp: 8579 Da, Obs: 8577 Da). Figure S6: Characterization of UbNHCH2CH2SeH: a) Analytical RP‐HPLC chromatogram 214 nm, b) ESI MS, c) Deconvoluted ESI MS (Selenol: Exp: 8671 Da, Obs: 8673 Da; Diselenide: Exp: 17340 Da, Obs: 17347 Da). Figure S7: Analysis of the reaction mixture of UbN3 and Selenocystamine: a) ESI MS, b) Deconvoluted ESI MS (UbCOOH: Exp: 8565 Da, Obs: 8563 Da; UbNHCH2CH2SeSeCH2CH2NH2: Exp: 8793 Da, Obs: 8793 Da). Figure S8: Analysis of UbSMeSNa: a) ESI MS, b) Deconvoluted ESI MS (UbCOOH: Exp: 8565 Da, Obs: 8565 Da; UbSMesNa: Exp: 8689 Da, Obs: 8689 Da); Analysis of the reaction mixture of UbSMeSNa and Selenocytsamine after three days: c) ESI MS, d) Deconvoluted ESI MS (UbCOOH: Exp: 8565 Da, Obs: 8565 Da; UbSMesNa: Exp: 8689 Da, Obs: 8689 Da; UbNHCH2CH2SeSeCH2CH2NH2: Exp: 8793 Da, Obs: 8793 Da). Figure S9: Stability of (UbNHCH2CH2Se)2: a) In reducing medium, pH 5, RT (analytical RP‐HPLC, 214 nm) b) In nonreducing medium, pH 5, RT (analytical RP‐HPLC, 214 nm), c) during storage of the lyophilized material at—20 °C (analytical RP‐HPLC, 214 nm, normalized for ease of comparison). Scheme S1: Proposed mechanism for hydrolysis of (UbNHCH2CH2Se)2 at pH 5 u [file PSC-31-e70037-s001.pdf]

Supplementary Information

Photocatalytic Diselenide Contraction as a Tool for Site-selective Isosteric Ubiquitylation

Herwig Weissinger, Moritz Urschbach, Luca Ferrari, Sascha Martens, Christian F. W. Becker

Contents

1 Materials and Methods 3
1.1 Analytical RP-HPLC 3
1.2 LCMS 3
1.3 Gel electrophoresis 4
1.4 Preparative RP-HPLC 4
1.5 Fmoc Solid Phase peptide synthesis 4
1.6 Photoreaction 6
2 Experimental procedures 6
2.1 Synthesis and deprotection of Tau F (291-326, K311U) 6
2.2 Synthesis of Tau selenalysine 9
2.3 Synthesis of ubiquitin selenocystamide 11
2.3.1 Synthesis of ubiquitin selenocystamine 15
2.3.2 Alternative Synthesis of $(UbNHCH_2CH_2Se)_2$ 16
2.3.3 Assessment of the stability of ubiquitin selenocystamine 18
2.4 PDC between ubiquitin and Tau-fragment 20
2.4.1 Without exclusion of oxygen 20
2.4.2 Strict exclusion of oxygen 21
2.4.3 Testing the influence of PTA concentration 23
2.4.4 PDC ubiquitin-tau using optimized conditions 25
2.5 Synthesis Ub-Tau heterodiselenides 27
2.5.1 Synthesis of Ub-SeSe-Tau from Tau-SeS-nitropyridyl and ubiquitin-SeH: 27
2.5.2 Photocatalyzed Synthesis of $UbNHCH_2CH_2SeSeTau$ from TauSeSnitropyridyl and $(UbNHCH_2CH_2Se)_2$: 28
2.5.3 Synthesis of ubiquitin-Tau heterodiselenide using equilibrium exchange reaction 30
2.6 Synthesis of selenylsulfides 31
2.6.1 Preparation of Selenylsulfides though exchange of diselenides with thiols 31

2.6.2 Preparation of selenylsulfides through substitution of 5-nitropyridyl selenylsulfides 33

3 Peptide sequences 35

3.1 Ub-Mxe-H7-CBD..39

3.2 Tau F (291-326, K311U) 39

1 Materials and Methods

DMF p.a., DCM p.a., ACN (HPLC grade), TIPS, Hydrazine monohydrate, DTNP, DPDS, TCEP were purchased from Sigma-Aldrich (Burlington, United States). TFA was purchased from Iris Biotech (Marktredwitz, Germany). Diethyl ether (HPLC grade) was purchased from VWR Chemicals (Radnor, United States). $[Ir(dF(CF_3)ppy)_2(dtbpy)]PF_6$ was purchased from BLDpharm (Shanghai, China). PTA was purchased from Thermo Fisher Scientific (Waltham, United States). Selenocystamine dihydrochloride 95% was purchased from AbCellera Biologics (Vancouver, Canada) and purified before use by dissolution in water and centrifugation to separate the insoluble contaminants. DTT was purchased from Carl Roth (Karlsruhe, Deutschland).

Water was obtained by Millipore Milli-Q purification system in combination with Miele Professional G7895 Ion exchange desalting system.

1.1 Analytical RP-HPLC

Unless noted otherwise all analytical RP-HPLC measurements were recorded on a Thermo Scientific Dionex UltiMate 3000 system using a Waters XBridge Protein BEH C4 Column with a particle size of 3.5 µm, pore size of 300 Å and the dimensions of 4.6×150 mm. All eluents were degassed in an ultrasonic bath *in vacuo*. Eluent A: 0.1% TFA in H_2O, Eluent B: 0.08% TFA in ACN. Gradient: 5% B for 5 min, 5–65% B in 30 min, 65–75% B in 3 min, 75% B for 3 min, 75–5% B in 4 min. Flow rate: 1 ml/min. Absorbance was recorded at 214 nm and 280 nm.

1.2 LCMS

LCMS was performed on a Waters Auto Purification System (3100 Mass Detector, 2545 Binary Gradient Module, 2767 Sample Manager and 2489, UV/Visible Detector) or Waters Arc HPLC system (SQ Detector 2 (Mass), Quaternary Solvent Manager-R, Sample Manger FTN-R and 2489 UV/Vis Detector). Mass spectra were acquired in positive ion mode utilizing electrospray ionization. Separation of the analytes was achieved on a Kromasil 300-5-C4 (dimensions: 4.6×50 mm) column at a flow rate of 1 ml/min and a gradient of 5-65% ACN+0.05% TFA in H_2O + 0.05% TFA. Masses were recorded in the range of 400-2000 Da (Or 400-3000 on the Waters Arc HPLC system).

1.3 Gel electrophoresis

Acrylamide gels were prepared using 7% Acrylamide in the stacking gel, 15% Acrylamide in the separating gel and 0.1% SDS in both. Samples were mixed with an equal volume of 500 mM Tris, 6% (w/v) SDS, 35% (v/v) glycerine, 3.55% (v/v) β-mercaptoethanol, 0.05% (w/v) bromophenol blue adjusted to pH 6.8 before loading. Whole cell samples were additionally incubated at 95°C for 5 min before loading. The running buffer was composed of 25 mM Tris, 200 mM glycine and 0.1% (w/v) SDS. All gels were run at a fixed voltage of 200 V for 40–50 min.

1.4 Preparative RP-HPLC

Purifications were carried out on a Waters Prep 150 LC HPLC-System, equipped with a Waters Fraction collector III and a Waters 2489 UV/Vis Detector.

Eluent A: 0.1% TFA in H_2O

Eluent B: 0.08% TFA in ACN

Gradient A: 5% B for 15 min, 5–25% B in 10 min, 25–40% B in 40 min, 40–85% B in 5 min, 85% B for 5 min, 85–5% B in 5 min.

Gradient B: 5% B for 6 min, 5–65% B in 46 min, 65–75% B in 8 min, 75% B for 10 min, 75–5% B in 5 min.

Gradient C: 5% B for 5 min, 5–65% B in 70 min, 65% B for 10 min, 65–5% B in 5 min.

Gradient D: 5% B for 10 min, 5–65% B in 60 min, 65% B for 10 min, 65–5% B in 5 min.

Gradient E: 5% B for 10 min, 5–50% B in 60 min, 50–65% B in 5 min, 65% B for 10 min, 65–5% B in 5 min.

Prior to injection, all samples were filtered using CHROMAFIL Xtra PES-20/25 0.20 µm syringe filters.

1.5 Fmoc Solid Phase peptide synthesis

Preparation 2-Chlorotrityl Hydrazide Resin: 2-CTC resin with a maximum loading capacity of 1.55 mmol/g (Novabiochem, 100–200 mesh) was washed consecutively with

DMF (3×5 mL), DCM (3×5 mL) and DMF (3×5 mL). The resin was allowed to swell in 1:1 DMF:DCM for 30 min. The resin was incubated in 10% (v/v) $N_2H_4 \cdot H_2O$ in DMF for 30 min. The solution was removed, the resin washed once with DMF and incubated again with 10% $N_2H_4 \cdot H_2O$ in DMF for 30 min. To reduce the loading of the resin, 60 mol% Boc-protected AA and 40 mol% Fmoc-protected AA was used. AA (4 eq.) and HBTU (3.8 eq.) were dissolved in DMF to a final AA concentration of 0.3 mol/l. After 2 min., DIPEA (8 eq.) was added. After 2 min., the solution was added to the resin and incubated for 1.5 h. Afterwards, the resin was drained and thoroughly washed with DMF, DCM and dried under reduced pressure.

Automated peptide synthesis (CEM Liberty PRIME)

Deprotection: The resin was treated with 20% piperidine in DMF under microwave irradiation. Deprotection was carried out for 1 min. at 90°C.

Coupling: The resin was treated with AA (6 eq., 0.5 M in DMF), DIC (14.4 eq., 2 M in DMF) and Oxyma (5 eq., 0.25 M in DMF). The coupling was carried out for 4 min. at 90°C under microwave irradiation. The resin was washed with DMF and the coupling step was repeated a second time.

Test cleavage: A small amount of resin (approx. 10 mg) was treated with 500 µl of 18:1:1 TFA:TIPS:H_2O for 2 h on a mixing wheel. The resin was washed with another 500 µl of the same mixture. The combined liquids were diluted to 10 ml using Et_2O. The samples were centrifuged at 4472 × g for 5 min, the supernatant was decanted, and the precipitate was dried under vacuum. The dried peptide was dissolved in 6 M Gdn·HCl and analysed using LCMS.

Cleavage (Tau): The peptide was cleaved from the solid support using TFA:TIPS/H2O (90:5:5, 1 mL/0.01 mmol loading capacity). The mixture was incubated with the resin on a mixing wheel for 3 h. The resin was washed with a small amount of TFA and the combined supernatants were precipitated from a 10-fold excess of cold Et_2O. The precipitate was collected via centrifugation at 4472 × g for 10 min and the supernatant discarded. The precipitate was resuspended in Et_2O, centrifuged again and the

supernatant discarded. The precipitate was dissolved in 1:1 ACN:H_2O and lyophilized to yield an off-white lyophilizate solid.

1.6 Photoreaction

A chip containing a LEDENGIN LED (450–500 nm with an emission maximum at 460 nm), powered at 3.3 V, 1 A was screwed into a heat sink. Above the heatsink, a 3D printed quadratic spacer with a height of 18 mm was mounted. The luminous intensity at the Eppendorf vial reached 16 mW/cm^2. On top of the spacer a 3D printed Eppendorf tube holder with a height of 38 mm was placed. The entire assembly was mounted on top of a magnetic stirrer by means of a clamp and wrapped with aluminium foil. The reaction mixture was placed inside a 1.5 ml Eppendorf tube alongside a 2×2 mm stir bar. The sample was put into the irradiation assembly at RT and the temperature rises to about 30°C during 5 min. of irradiation.

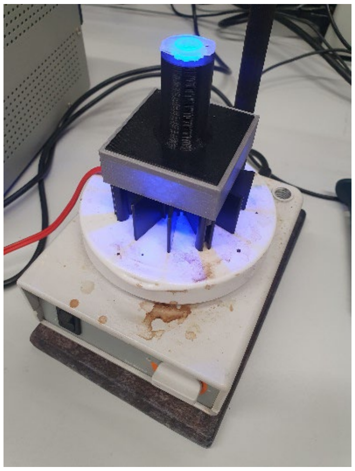

Figure 1: Picture of the irradiation Setup during use with the foil removed for visibility

2 Experimental procedures

2.1 Synthesis and deprotection of Tau F (291-326, K311U)

The Tau F fragment was needed as model substrate for photocatalyzed diselenide contraction reaction. Furthermore, ubiquitylated Tau F could be used in protein aggregation studies to further elucidate the influence of PTMs on Alzheimer’s disease. The fragment with the primary structure

^{291}GSKDNIKHVPGGGSVQIVYU(Mob)PVDLSKVTSKC(Acm)GSLG^{326} was synthesized using Fmoc SPPS at a scale of 0.1 mmol as described in **9.7**. The position for the lysine to selenocysteine point mutation was chosen because Tau F protein is frequently ubiquitylated at this position, especially in Alzheimer's disease patients[44]. After TFA/TIPS/H2O mediated simultaneous cleavage from the resin and removal of all protecting groups other than methoxybenzyl (Mob) and acetamidomethyl (Acm), the product was dissolved, lyophilized to yield 350 mg of crude yellowish solid. The protecting groups Mob and Acm were chosen because of their orthogonality. To prevent formation of selenylsulfides, conditions for the selective deprotection of Mob were tested (**Table 1**).

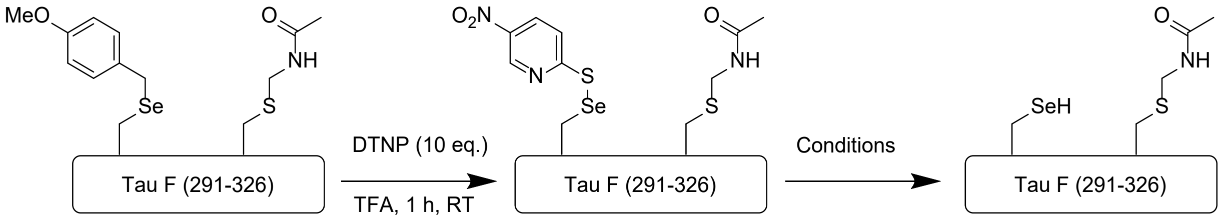

Table 1: Screening of conditions for selective deprotection of 5-nitropyridyl.

Conditions	Results
3 eq. DTT in TFA, 30 min, RT	No reaction observed
5 eq. ascorbic acid in 33% H_2O/ 66% TFA, 30 min, RT	No reaction observed
27 eq. Na-ascorbate in H_2O, pH 4.5, 50 h	Complete reaction, partial Acm removal
60% TFA 20% DMSO 20% 6 M Gdn·HCl, 0.1 M Hepes, RT, 3.5 h	Complete removal of both protecting groups
**15 eq. DTT in H_2O, 4.5 h, RT**	**Complete and selective removal of the 5-nitropyridyl protecting group**
5 eq. Na-ascorbate in H_2O, pH 7, 4.5 h, RT	Mixture of product and starting material
2 eq. TCEP in H_2O, RT	Unselective deprotection
5 eq. PhSH in 80% H_2O 20% ACN, 2.5 h, RT	-SeSPh formed, unselective deprotection

Of the tested conditions, a two-step procedure consisting of conversion to the 5-nitropyridyl selenylsulfide using DTNP in TFA and subsequent deprotection using DTT in

H_2O proved most successful in terms of conversion and selectivity when analysed by LCMS. After the first step, the crude product was precipitated in Et_2O and washed to remove residual TFA. The Et_2O was removed under vacuum and the product was used in the second step without purification. When DTT was added, the mixture became bright yellow due to formation of 5-nitropyridyl thiolate. Purification via preparative RP-HPLC

yielded 25.9 mg of Tau F (291-326, K311U) diselenide, henceforth designated as $(TauSe)_2$ (**Figure 2).**

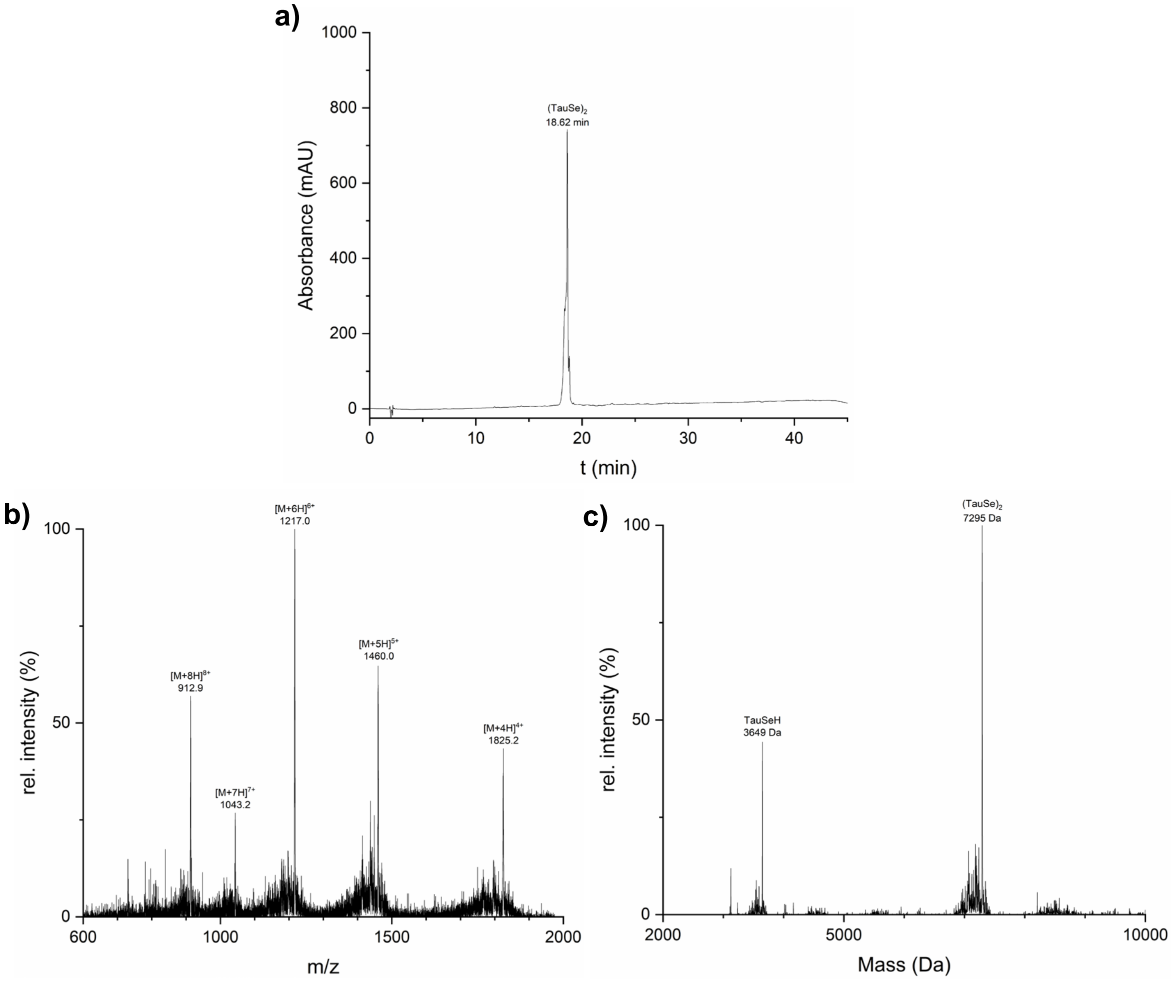

Figure 2: Characterization of Tau F (291-326, K311U): a) Analytical RP-HPLC chromatogram 214 nm, b) ESI MS, c) Deconvoluted ESI MS (Selenol: Exp: 3649 Da, Obs: 3649 Da; Diselenide: Exp: 7296 Da, Obs: 7295 Da).

2.2 Synthesis of Tau selenalysine

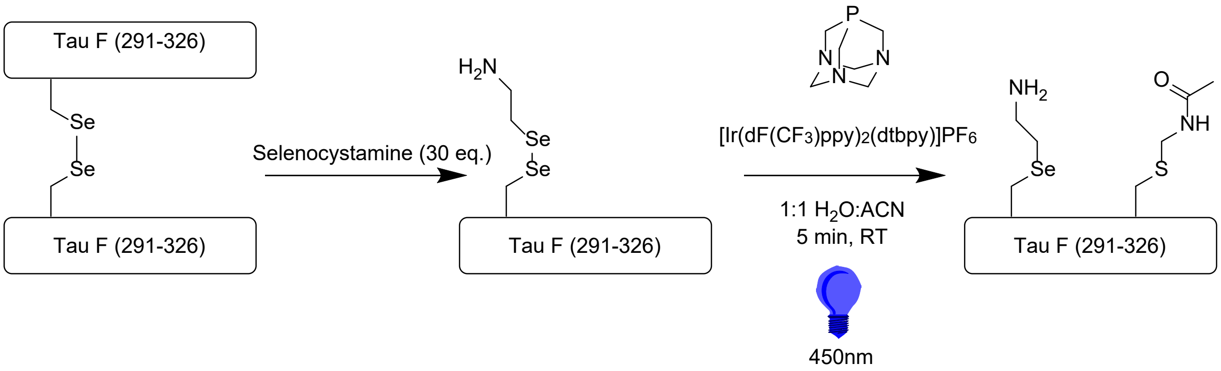

$(TauSe)_2$ (1mg, 0.14 μmol) was dissolved in 100 μl of 1:1 ACN:H_2O. Selenocystamine dihydrochloride solution in water (13 μl, 0.1 mg/μl, 4.1 μmol, 30 eq.) was added. $(Ir[dF(CF_3)ppy]_2(dtbpy))PF_6$ solution in ACN (15μl, 0.5 mg/ml, 7.5 μg, 6.7 nmol, 5 mol%) was added. PTA solution in water (5 μl, 0.11 mg/μl, 0.55 mg, 3.5 μmol, 25.5 eq.) was added. A stir bar was added, and the reaction mixture was irradiated (5 min, 460 nm). The reaction mixture was analysed using LCMS, there were substantial amounts of unreacted $TauSeSeCH_2CH_2NH_2$ (3771 Da) left. Repeated irradiation did not improve conversion, so additional PTA solution (5 μl, 0.11 mg/μl, 3.5 μmol, 25.5 eq.) was added.

The reaction mixture was irradiated again. LCMS confirmed complete consumption of the starting material. An approximate yield of 60% is calculated by integration of the RP-

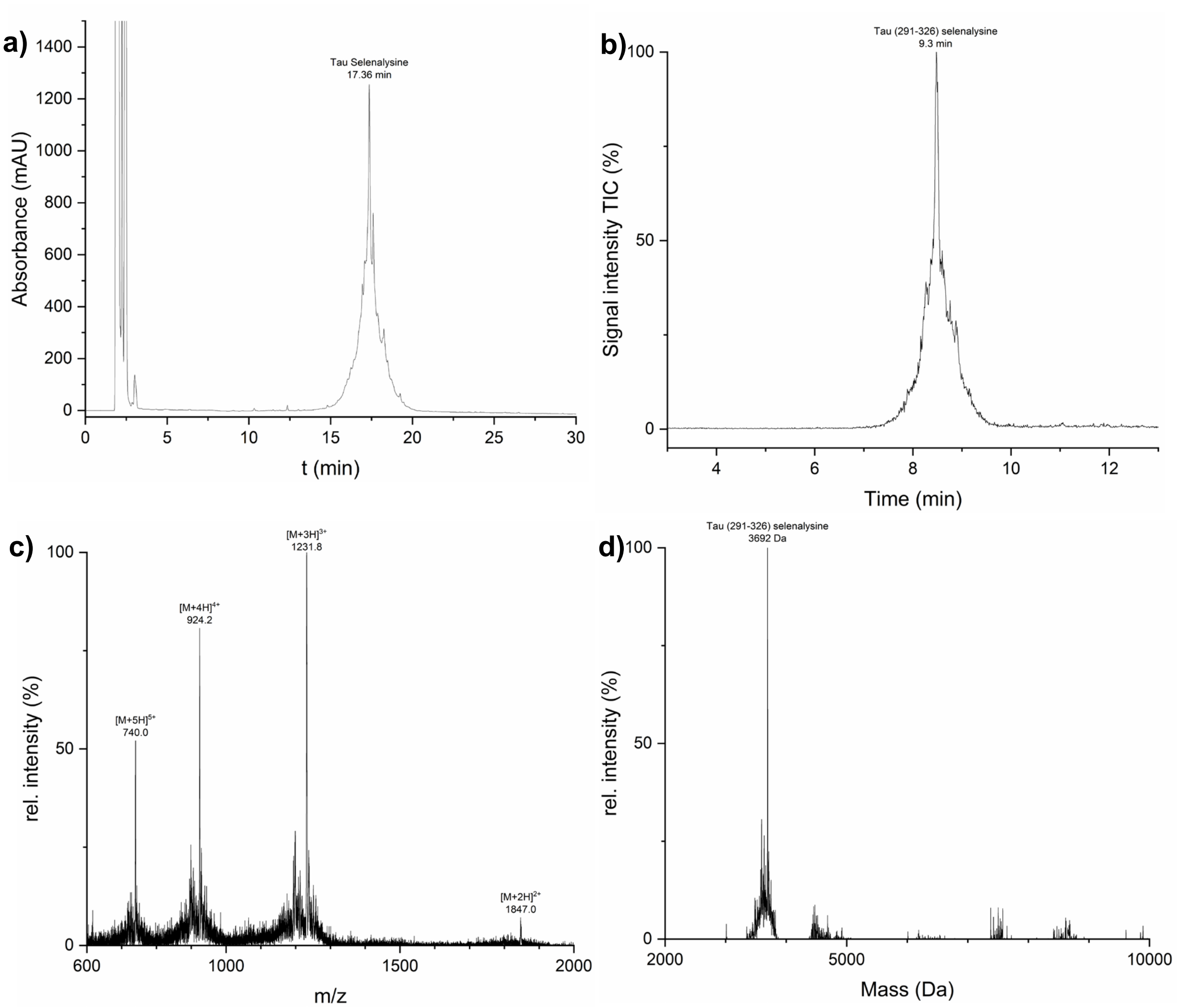

Figure 3: Characterization of crude Tau (291-326) selenalysine: a) Analytical RP-HPLC chromatogram at 214 nm, b) LCMS TIC chromatogram, c) ESI MS of peak at 8.5 min, d) Deconvoluted ESI MS of peak at 8.5 min (Exp: 3692 Da, Obs: 3692 Da).

HPLC peak at 214 nm.

2.3 Synthesis of ubiquitin selenocystamide

Inoculation:

The preculture was prepared in 2×500 mL Erlenmeyer flasks containing sterile 2YT medium (200 ml, Tryptone 16 g/l; Yeast extract 10 g/l; NaCl 5 g/l) with ampicillin (100 mg/l). Each flask was inoculated with *E. Coli* BC21(DE3) Gold equipped with a pTXB1

vector encoding for the Ubiquitin-Mxe Gyr A-H7-CBD fusion protein using a flame disinfected toothpick. The broth was incubated overnight at 37°C at 180 rpm shaking. After 16 h of incubation the optical density at 600 nm (OD_{600}) of the precultures was measured to be 3.2. To each of the 5 l Erlenmeyer flasks was added 2 ml of 100 mg/l Ampicillin solution. The expression was performed by inoculating 2 x 2 l of 2YT medium containing ampicillin (100 mg/l) with the preculture (~100 ml) to an OD_{600} ~0.2. The cultures were incubated at 37°C and 180 rpm for 2.5 h or until the OD_{600} reaches ~0.6. To induce the expression of the fusion construct, to both of the flasks Isopropyl-β-D-thiogalactopyranosid (IPTG) was added to a final concentration of 1 mmol/l. The flasks were incubated for 2.5 h using the same conditions as above and samples are taken at 1, 2 and 2.5 h.

Harvesting:

The cultures were centrifuged at 7674 × g for 25 min. The supernatant was discarded, and the collected cells were kept at -80°C until the next day. The cells were resuspended in 160 ml TBS. The solution was cooled on ice and the cells were disrupted using ultrasonification (10 min., 500 W at 20 kHz and 60% amplitude, ON-OFF cycle: 15 s On,

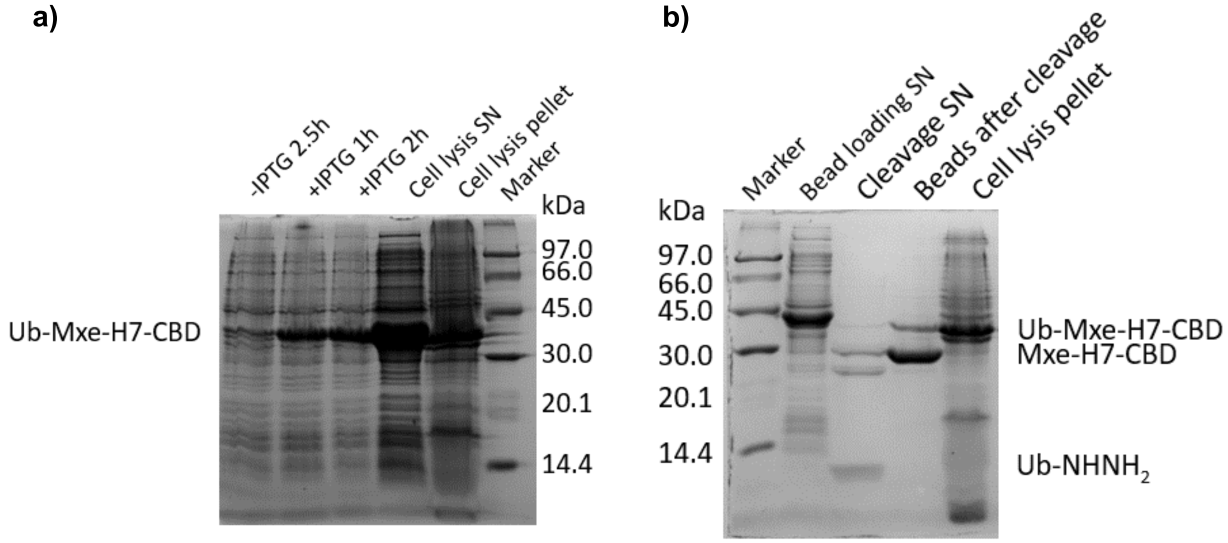

Figure 4: Gel electrophoresis a) during expression b) and during purification.

45 s Off). The disrupted cells were centrifuged at 76608 × g for 30 min. The supernatant was distributed onto 4×5 ml of chitin beads and incubated on the tube roller for 2 h. Afterwards, the supernatant was removed but kept at 4°C until complete loading was

confirmed using gel electrophoresis. The beads were washed two times using 35 ml TBS each and the washings were discarded.

Cleavage:

The cleavage solution was prepared by dissolving hydrazine monohydrate (4.3 ml, 2% v/v final concentration) and DTT (1.08 g, 50 mM/l) in 140 ml of distilled water. A sample of the beads was taken, and the cleavage solution was distributed onto the beads and incubated on the tube roller for 3 d at RT. Using gel electrophoresis, it was observed that the cleavage from the beads was incomplete after 3 days, so the beads were allowed to react for one more day on the roller. One Day later, gel electrophoresis was repeated, and the cleavage was mostly complete. The beads were allowed to settle, and the supernatant was transferred. The four vessels with beads were washed with two times 10 ml of TBS which was also added to the supernatant.

Purification:

The collected supernatant was filtered through a 0.22 µm PES syringe filters and purified via preparative RP-HPLC (Gradient A; Column: Kromasil 300-10-C4, 21.2×250 mm at 60°C) where the product eluted between 36-42 min. The fractions were analysed using MS, pooled into a pure main fraction and two side fractions of lesser purity and lyophilized.

The weight of the main fraction was 23.1 mg, side fraction 1 was 16.4 mg and the side fraction 2 was 3.0 mg.

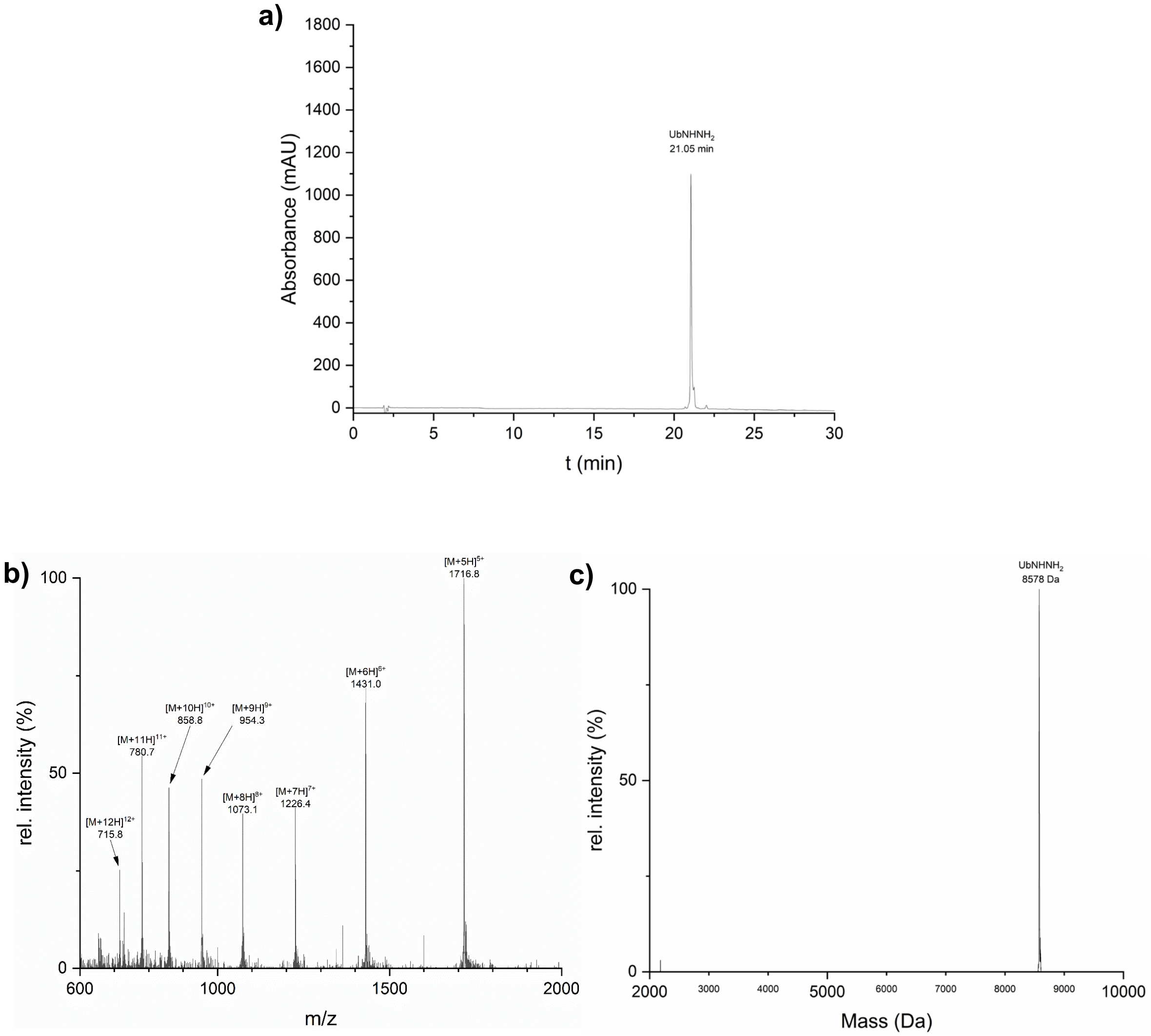

Figure 5: Characterization of $UbNHNH_2$: a) Analytical RP-HPLC chromatogram 214 nm, b) ESI MS, c) Deconvoluted ESI MS (Exp: 8579 Da, Obs: 8577 Da).

2.3.1 Synthesis of ubiquitin selenocystamine

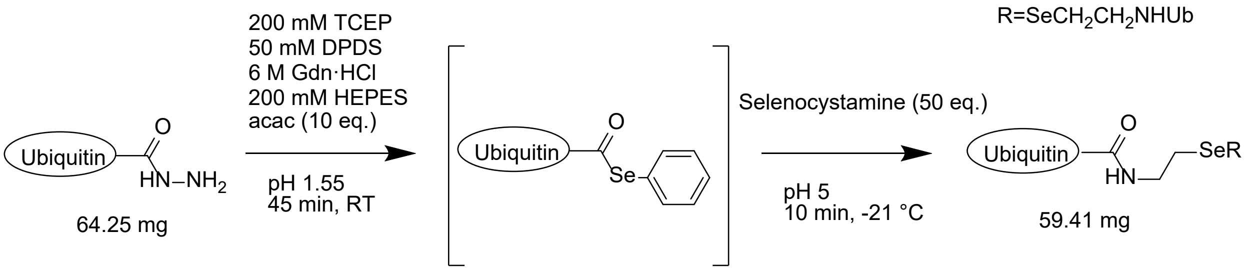

Ub-NHNH2 (64.25 mg, 7.5 µmol) was dissolved in freshly prepared reaction buffer (7.75 mL, 200 mM HEPES, 6 M Gdn·HCl, 200 mM TCEP, 50 mM DPDS). Some of the DPDS remains undissolved. The pH was adjusted to 1.55 using conc. HCl. Acetylacetone (7.5 µl, 7.7 mg, 76 µmol, 10 eq.) was added and the mixture was stirred at room temperature for 45 min. An ice-salt bath was prepared, and the reaction mixture was cooled to -20°C. Selenocystamine dihydrochloride solution (119 mg in 400 µl H_2O, 0.37 mmol, 50 eq.) was added and the pH was adjusted to 5.00. Special care must be taken to avoid warming the solution during pH adjustment, as the product hydrolyses rapidly at pH 5 and RT. After 10 min. of reaction time at pH 5 the solution was filtered through a piece of cotton using a syringe to remove the bulk of the undissolved DPDS. The remaining DPDS was extracted using 5×1 ml of Et_2O. In order to avoid warming the solution, the aqueous phase was frozen using dry ice/acetone, the Et_2O was poured off and the mixture was allowed to thaw in a salt/ice bath. The resulting solution was filtered through a 0.2 µm syringe filter. The vessel and syringe filter were washed using distilled water and the combined fractions were purified using preparative RP-HPLC (Gradient A; Column: Macherey-Nagel Nucleodur 300-5 C4ec, 21×250 mm) where the product elutes between 56 – 64 min. The fractions were analysed using MS and combined and lyophilized to yield the product as a mixture of selenol and diselenide (59.41 mg, 6.8 µmol

(calculated as the selenol), 91% yield) as a colourless lyophilizate. During storage at -20°C the product is gradually oxidized to the diselenide.

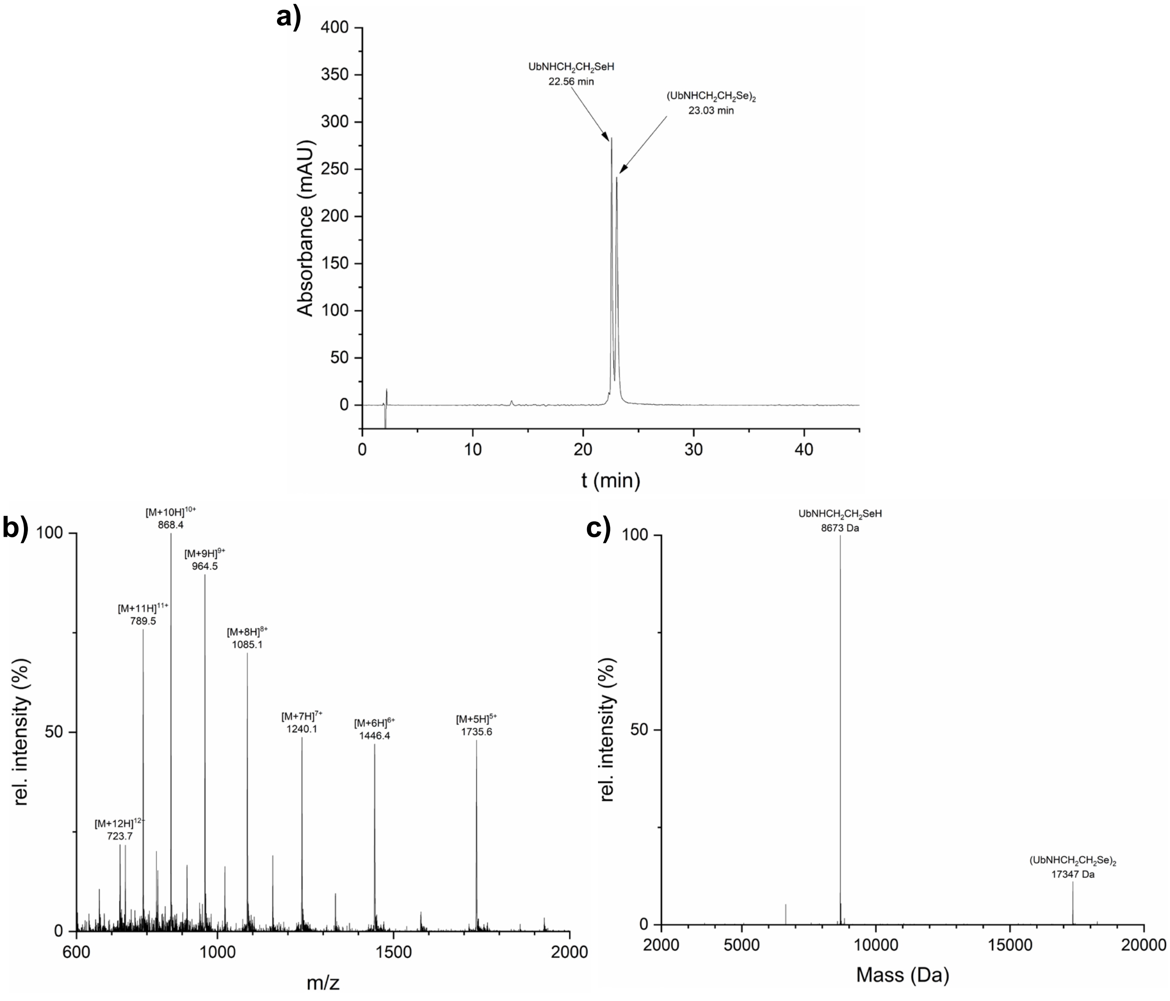

Figure 6: Characterization of UbNHCH2CH2SeH: a) Analytical RP-HPLC chromatogram 214 nm, b) ESI MS, c) Deconvoluted ESI MS (Selenol: Exp: 8671 Da, Obs: 8673 Da; Diselenide: Exp: 17340 Da, Obs: 17347 Da).

2.3.2 Alternative Synthesis of $(UbNHCH_2CH_2Se)_2$

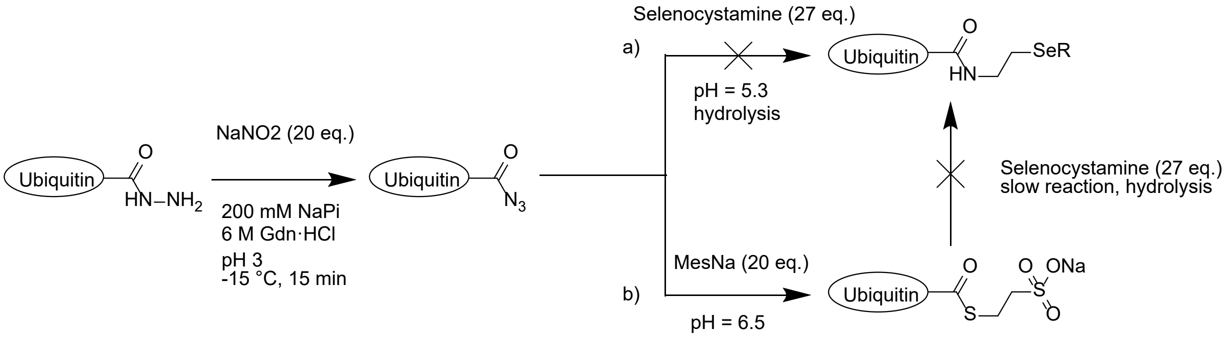

a) $UbNHNH_2$ (1 mg, 0.12 µmol) was dissolved in 56 µl of 6 M Gdn·HCl and 0.2 M NaH_2PO_4 adjusted to pH 3. The solution was cooled in a salt/ice bath. Sodium nitrite (18 µl, 1 mg/ml, 18 µg, 0.26 µmol, 2.2 eq.) solution was added. After 15 min, aqueous selenocystamine dihydrochloride solution (10 µl, 100 mg/ml, 1 mg, 3.13 µmol, 26 eq.) was added. The pH was adjusted to 5.3 and the reaction was allowed to continue for another 20 min in the salt/ice bath. LCMS revealed mostly hydrolysis of the azide but also small amounts $(UbNHCH_2CH_2Se)_2$.

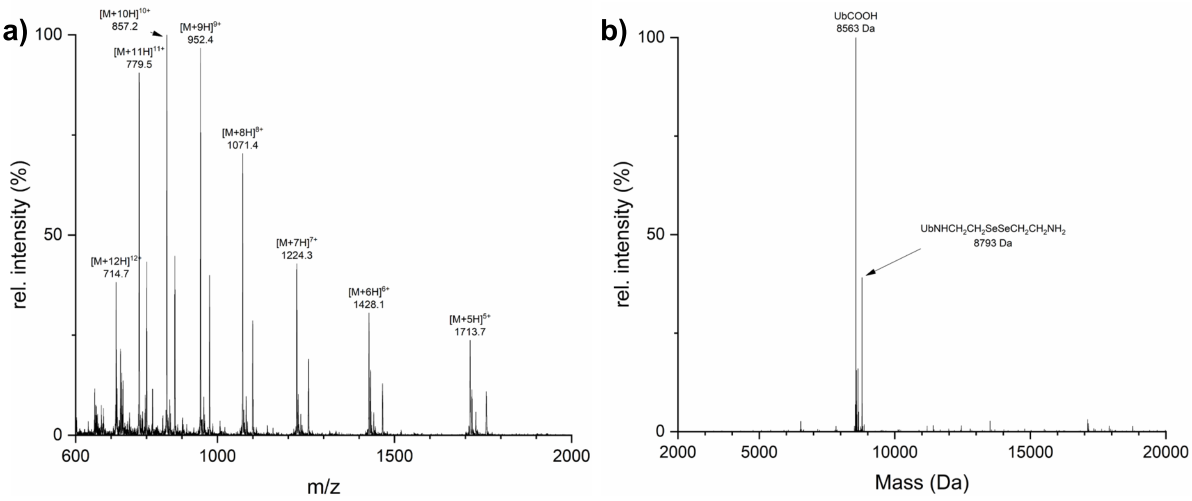

Figure 7: Analysis of the reaction mixture of UbN_3 and Selenocystamine: a) ESI MS, b) Deconvoluted ESI MS (UbCOOH: Exp: 8565 Da, Obs: 8563 Da; $UbNHCH_2CH_2SeSeCH_2CH_2NH_2$: Exp: 8793 Da, Obs: 8793 Da).

b) $UbNHNH_2$ (1 mg, 0.12 µmol) was dissolved in 56 µl of 6 M Gdn·HCl and 0.2 M NaH_2PO_4 adjusted to pH 3. The solution was cooled in a salt/ice bath to -20°C. Sodium nitrite (18 µl, 1 mg/ml, 18 µg, 0.26 µmol, 2.2 eq.) solution was added. After 15 min of reaction time, MesNa solution in 6 M Gdn·HCl and 0.2 M NaH_2PO_4 (50 µl, 9.5 mg/ml, 2.89 µmol, 24 eq.) was added. The pH was adjusted to 6.56 and the reaction was allowed to continue in the salt/ice bath for 10 min, then for 20 min at RT. LCMS confirmed complete conversion to the MesNa thioester as well as some hydrolysis (**Figure 8**). Selenocystamine dihydrochloride solution (10 µl, 100 mg/ml, 1 mg, 3.13 µmol, 26 eq.) was added. After 50 min of reaction time at RT, no change was visible in LCMS. After 3 days of reaction time at RT another LCMS was measured, showing some ubiquitin selenocystamine, but mostly

unreacted starting material as well as considerable amounts of hydrolysis (**Figure 8**).

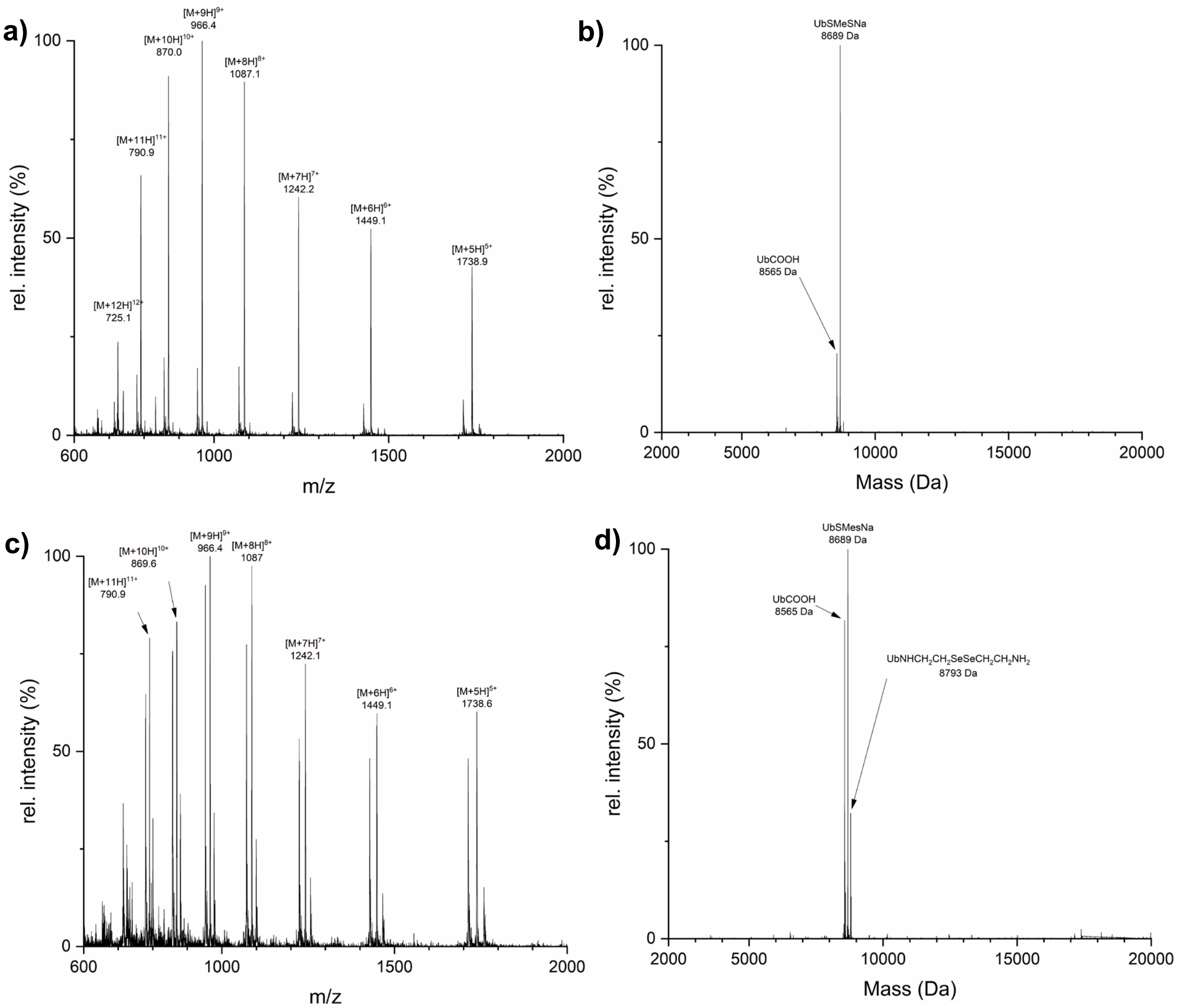

Figure 8: Analysis of UbSMeSNa: a) ESI MS, b) Deconvoluted ESI MS (UbCOOH: Exp: 8565 Da, Obs: 8565 Da; UbSMesNa: Exp: 8689 Da, Obs: 8689 Da); Analysis of the reaction mixture of UbSMeSNa and Selenocytsamine after three days: c) ESI MS, d) Deconvoluted ESI MS (UbCOOH: Exp: 8565 Da, Obs: 8565 Da; UbSMesNa: Exp: 8689 Da, Obs: 8689 Da; $UbNHCH_2CH_2SeSeCH_2CH_2NH_2$: Exp: 8793 Da, Obs: 8793 Da).

2.3.3 Assessment of the stability of ubiquitin selenocystamine

As hydrolysis was a common issue observed during the synthesis of ubiquitin selenocystamine, the stability of this product under different conditions was evaluated in order to control this side reaction. The stability was evaluated by preparing a saturated solution of (<5 mg/ml) ubiquitin selenocystamine in 1 M Gdn·HCl, 0.02 M NaH_2PO_4 with (**Figure 9, a**) and without 0.75 M TCEP (**Figure 9, b**). The undissolved protein was

removed by centrifugation, the pH was adjusted to 5 and analytical RP-HPLC was measured at 0.5, 1.5, 2.5 and 3.5 h. To confirm stability of the lyophilized product at –20°C, RP-HPLC was measured directly after synthesis and after two months of storage (**Figure 9, c**).

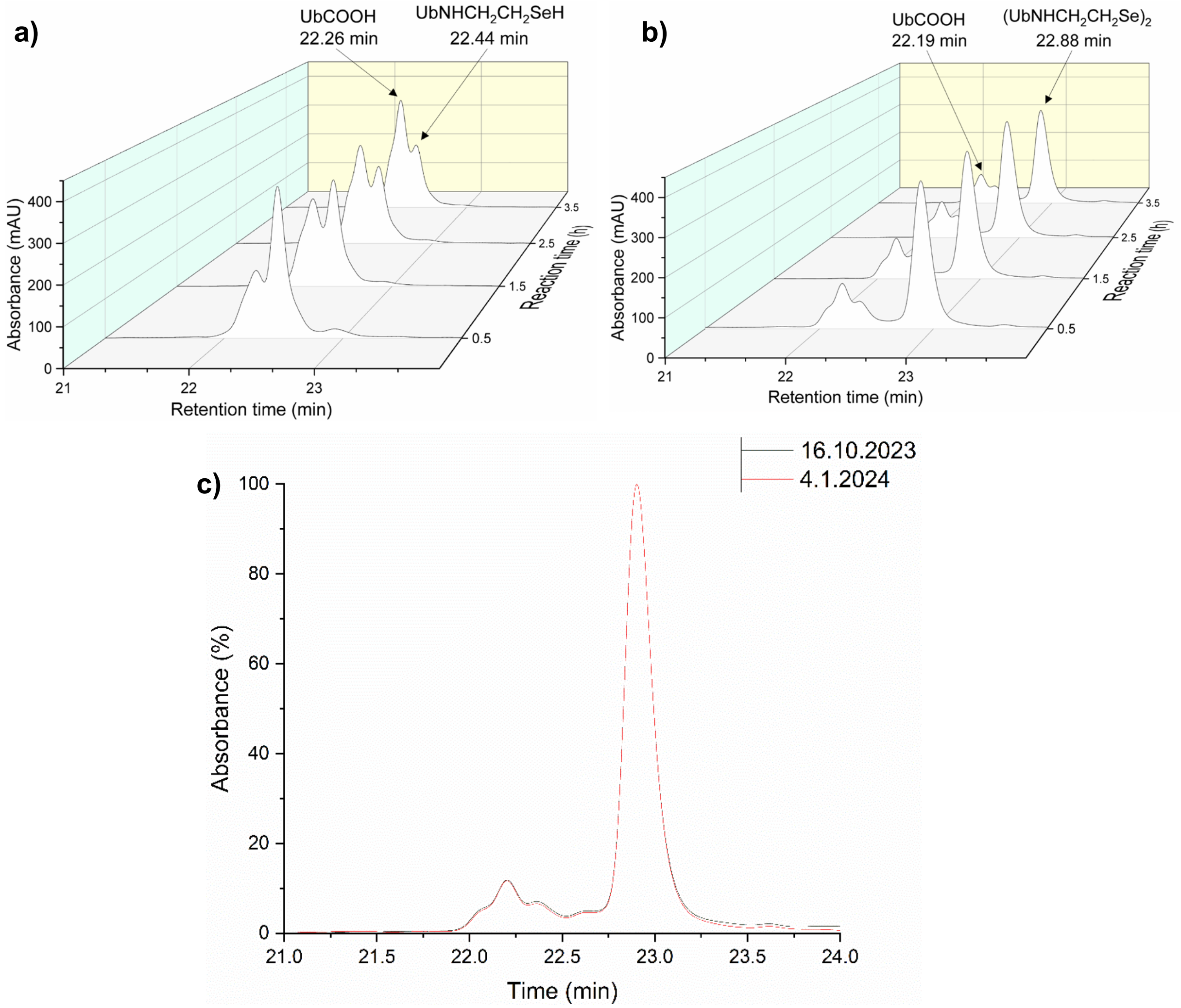

Figure 9: Stability of $(UbNHCH_2CH_2Se)_2$: a) In reducing medium, pH 5, RT (analytical RP-HPLC, 214 nm) b) In nonreducing medium, pH 5, RT (analytical RP-HPLC, 214 nm), c) during storage of the lyophilized material at - 20°C (analytical RP-HPLC, 214 nm, normalized for ease of comparison).

Based on the findings above a mechanism for the hydrolysis is proposed involving a selenoester intermediate. The mechanism explains the stability in the absence reducing agent as the rearrangement relies on nucleophilic attack of the selenate, which is considerably more nucleophilic than the diselenide (**Scheme 1**).

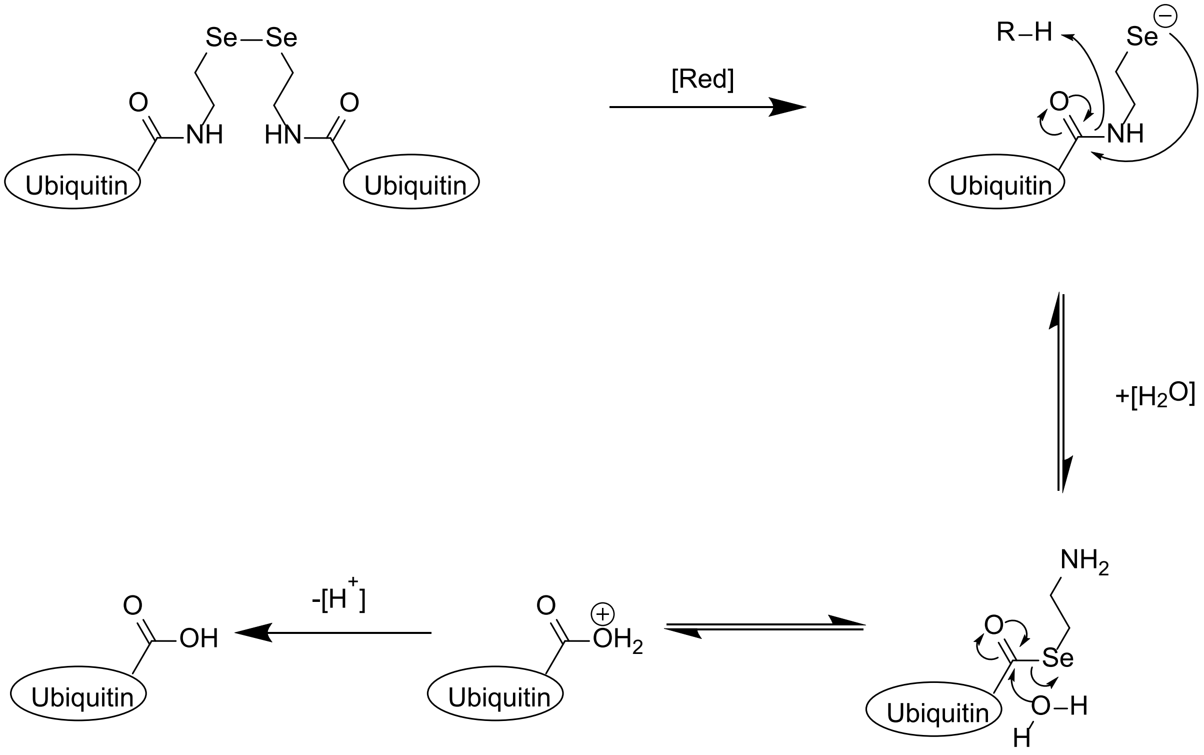

Scheme 1: Proposed mechanism for hydrolysis of $(UbNHCH_2CH_2Se)_2$ at pH 5 under reducing conditions.

2.4 PDC between ubiquitin and Tau-fragment

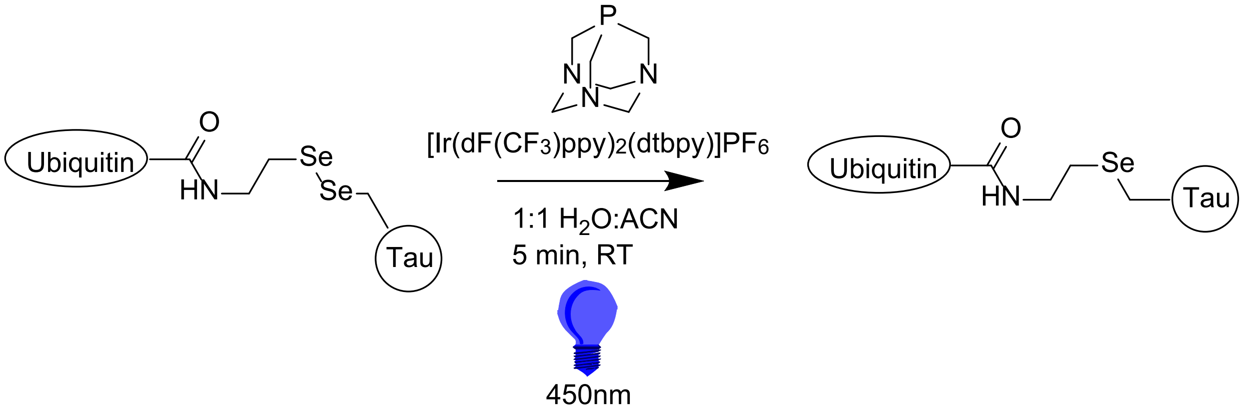

2.4.1 Without exclusion of oxygen

$UbNHCH_2CH_2SeSeTau$ (0.5 mg, 40.6 nmol) was weighed out and dissolved in 40 µl of water and $(Ir[dF(CF_3)ppy]_2(dtbpy))PF_6$ solution (34 µl, 0.5 mg/ml, 17 µg, 15 nmol, 37 mol%) in ACN was added. PTA (4.1 µl, 0.0125 mg/ml, 51.25 µg, 0.33 µmol, 8 eq.)

solution was added. The mixture was irradiated (5 min, 460 nm) and LCMS was measured.

No selenoether was produced, solely oxidized species were found.

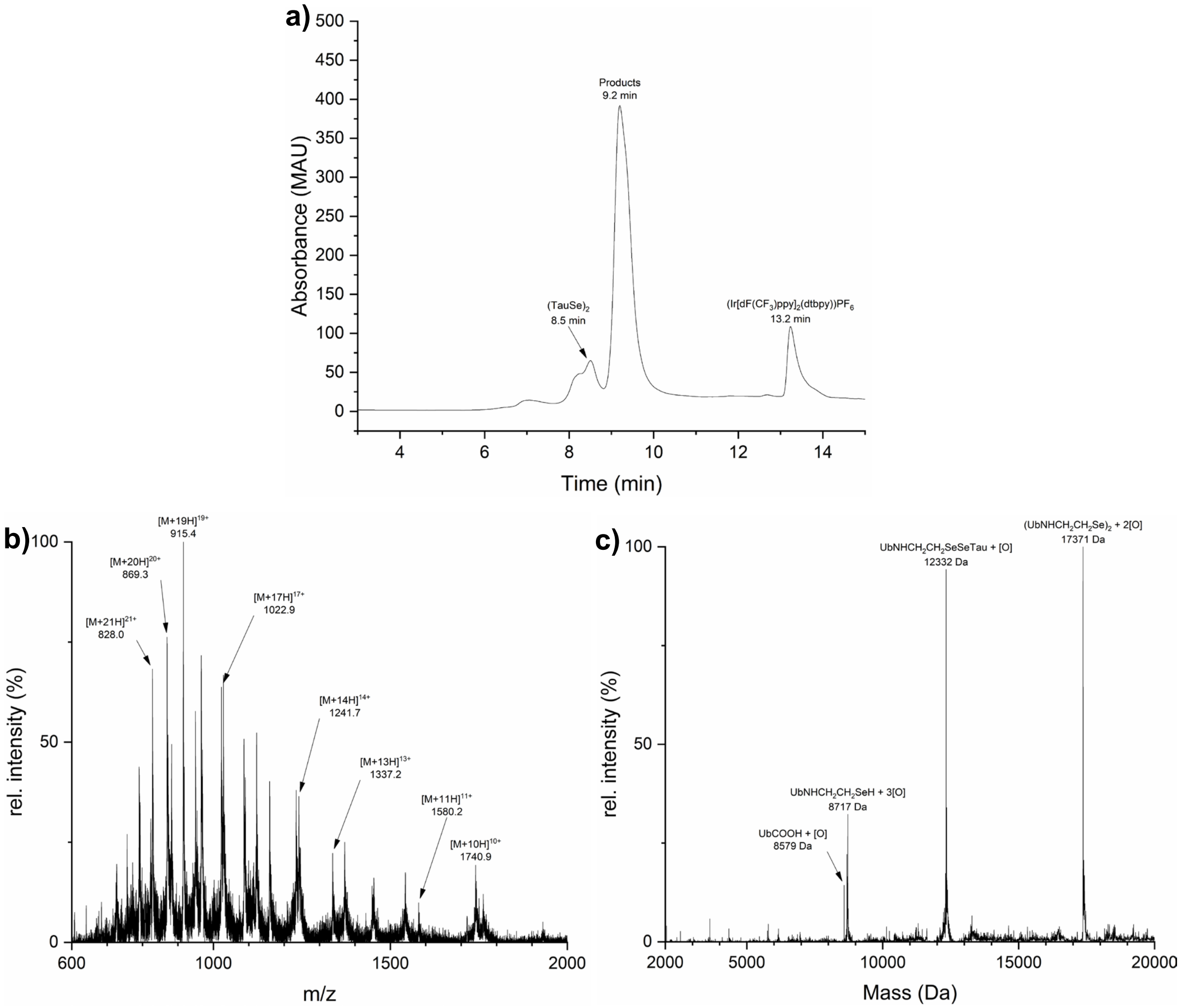

Figure 10: Analysis of reaction mixture of the PDC reaction between $(UbNHCH_2CH_2Se)_2$ and $(TauSe)_2$: a) LCMS at 214 nm , b) ESI MS of peak at 9.2 min, c) Deconvoluted ESI MS of peak at 9.2 min (UbCOOH + [O]: Exp: 3692 Da, Obs: 3692 Da; $UbNHCH_2CH_2SeH$ + 3[O]: Exp: 8719 Da, Obs: 8717 Da; $UbNHCH_2CH_2SeSeTau$ + [O]: Exp: 12334 Da, Obs: 12332 Da; $(UbNHCH_2CH_2Se)_2$ + 2[O]: Exp: 17372 Da, Obs: 17371 Da).

2.4.2 Strict exclusion of oxygen

All solutions were freshly prepared using degassed solvents (three of cycles Freeze-Pump-Thaw) and continuously flushed with N_2 when open. $UbNHCH_2CH_2SeSeTau$ (0.3 mg, 24.4 nmol) was weighed out and dissolved in 21 µl of water and $(Ir[dF(CF_3)ppy]_2(dtbpy))PF_6$ solution (7 µl, 1 mg/ml, 7 µg, 6.24 nmol, 26 mol%) in ACN was added. 17 µl of ACN and PTA solution (3 µl, 10 mg/ml, 30 µg, 0.19 µmol, 8 eq.) were

added. A stir bar was added, the vial was filled with Argon and the mixture was irradiated (5 min, 460 nm). LCMS confirmed complete suppression of oxidation, but almost no product was formed.

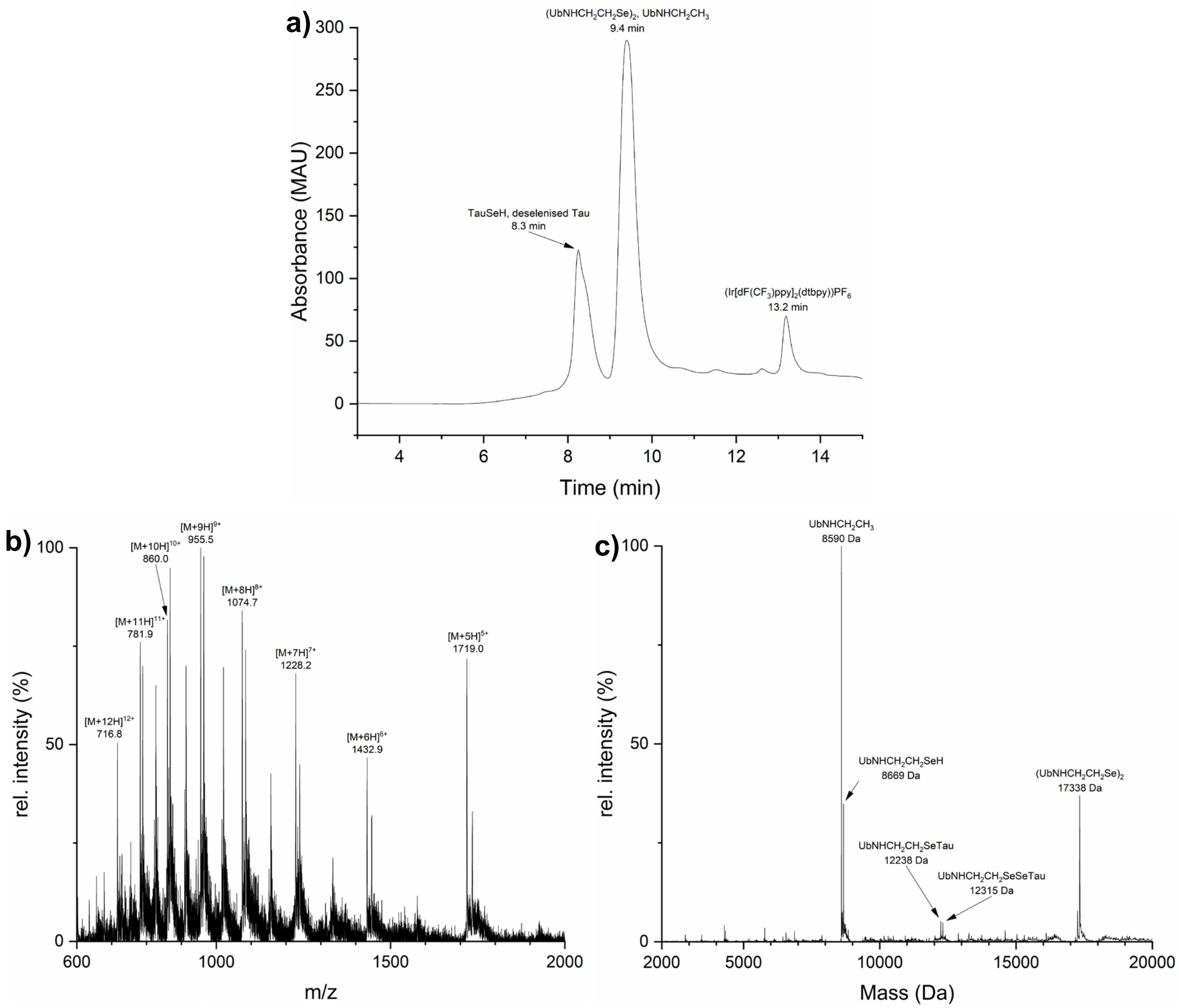

Figure 11: Analysis of reaction mixture of the PDC reaction between $(UbNHCH_2CH_2Se)_2$ and the $(TauSe)_2$ under strict exclusion of oxygen: a) LCMS at 214 nm , b) ESI MS of peak at 9.4 min, c) Deconvoluted ESI MS of peak at 9.4 min ($UbNHCH_2CH_3$: Exp: 8592 Da, Obs: 8590 Da; $UbNHCH_2CH_2SeH$: Exp: 8671 Da, Obs: 8669 Da; $UbNHCH_2CH_2SeTau$: Exp: 12239 Da, Obs: 12238 Da; $UbNHCH_2CH_2SeSeTau$: Exp: 12318 Da, Obs: 12315 Da; $(UbNHCH_2CH_2Se)_2$: Exp: 17340 Da, Obs: 17338 Da).

2.4.3 Testing the influence of PTA concentration

$(UbNHCH_2CH_2Se)_2$ (0.75 mg, 43.25 nmol) and $(TauSe)_2$ (2 mg, 0.274 µmol, 6.3 eq.) were weighed out and dissolved in 1 ml of 1:1 ACN:H_2O. The solution was incubated for 2 h in the ultrasound bath. LCMS confirmed complete equilibration of the diselenide mixture. The mixture was lyophilized, dissolved in 80 µl of 1:1 ACN:H_2O and an aliquot of 40 µl was moved to a different vial. To each of the vials, a solution of $(Ir[dF(CF_3)ppy]_2(dtbpy))PF_6$ in ACN (10 µl, 1 mg/ml, 10 µg, 8.91 nmol, 41 mol%) and was added. A solution of PTA was added to the first vial (2 µl, 50 mg/ml, 0.1 mg, 0.64 µmol, 29 eq.) and the second (4 µl, 50 mg/ml, 0.2 mg, 1.28 µmol, 58 eq.) added. Both reaction mixtures were irradiated (5 min, 460 nm). LCMS revealed incomplete conversion of the starting material when 29 eq. of PTA were used and complete conversion in the case of 58 eq.

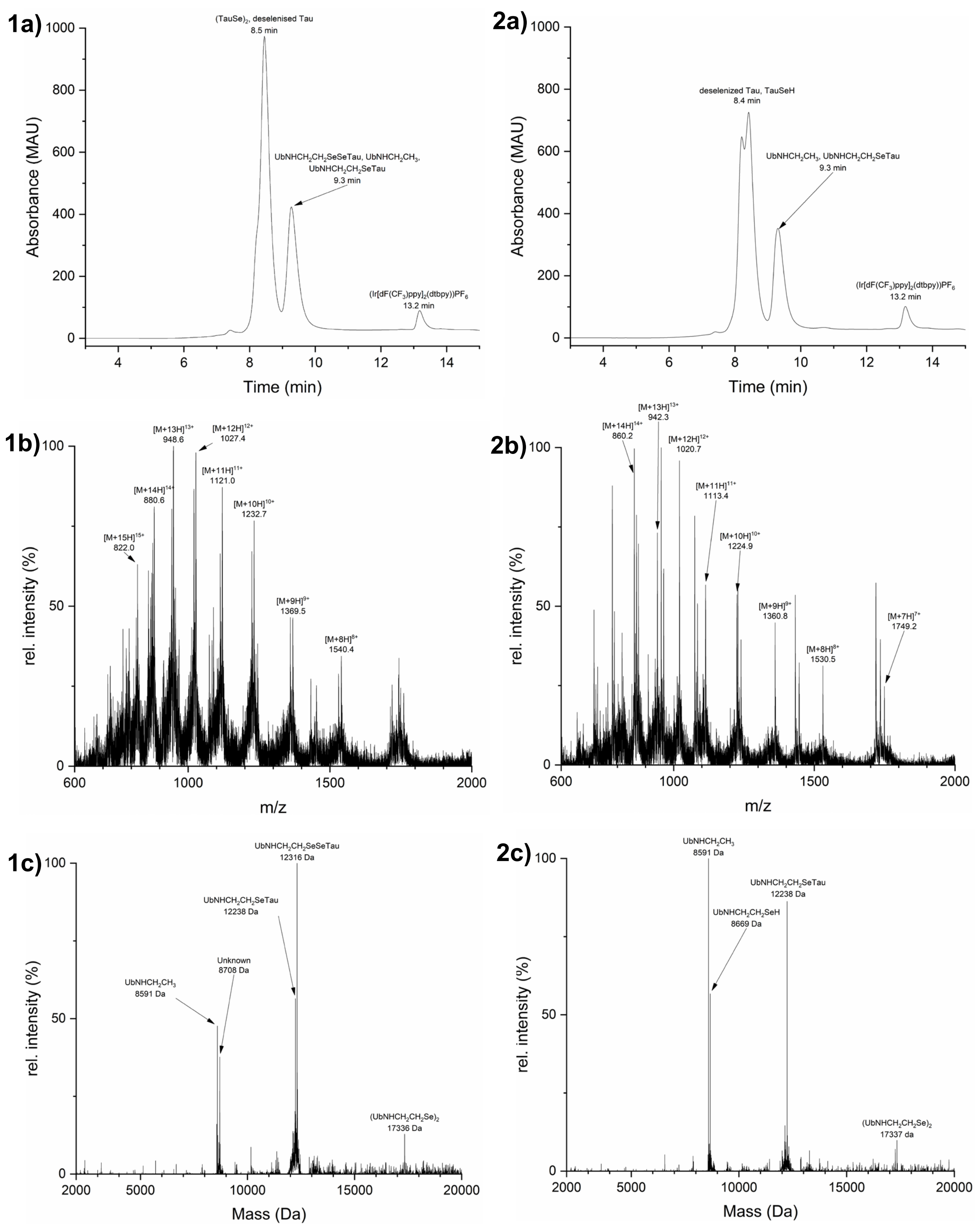

Figure 12: Comparison between 4 eq. (1a-c) and 8 eq. (2a-c) of PTA compared to the total diselenide content for the PDC reaction of $(UbNHCH_2CH_2Se)_2$ and $(TauSe)_2$: a) LCMS at 214 nm, b) ESI MS of peak at 9.3 min, c) Deconvoluted ESI MS of peak at 9.3 min ((1c): $UbNHCH_2CH_3$: Exp: 8592 Da, Obs: 8591 Da; $UbNHCH_2CH_2SeTau$: Exp: 12239 Da, Obs: 12238 Da; $UbNHCH_2CH_2SeSeTau$: Exp: 12318 Da, Obs: 12316 Da; $(UbNHCH_2CH_2Se)_2$: Exp: 17340 Da, Obs: 17336 Da, (2c): $UbNHCH_2CH_3$: Exp: 8592 Da, Obs: 8591 Da; $UbNHCH_2CH_2SeH$: Exp: 8671 Da, Obs: 8669 Da; $UbNHCH_2CH_2SeTau$: Exp: 12239 Da, Obs: 12238 Da; $(UbNHCH_2CH_2Se)_2$: Exp: 17340 Da, Obs: 17337 Da).

2.4.4 PDC ubiquitin-tau using optimized conditions

$UbNHCH_2CH_2SeSeTau$ (0.5 mg, 37.5 nmol) and $(TauSe)_2$ (1 mg, 0.137 µmol, 3.5 eq.) were weighed out and dissolved in 38 µl of H_2O and 28 µl of ACN. A solution of $(Ir[dF(CF_3)ppy]_2(dtbpy))PF_6$ in ACN (11.7 µl, 1 mg/ml, 11.7 µg, 10.4 nmol, 28 mol%) and a solution of PTA (3 µl, 69 mg/ml, 0.21 mg, 1.32 µmol, 35 eq.) were added. The reaction mixture was irradiated for 5 min. LCMS indicated full consumption of the starting material and appreciable amounts of the product selenoether. The reaction was repeated three more times, and the products were pooled. The mixture was purified via preparative RP-HPLC (Gradient C, MZ-PerfectSil 300-10-C4, 10×250 mm, 60°C). The product eluted between 43 and 44 min. The fractions were analysed using MS and all fractions

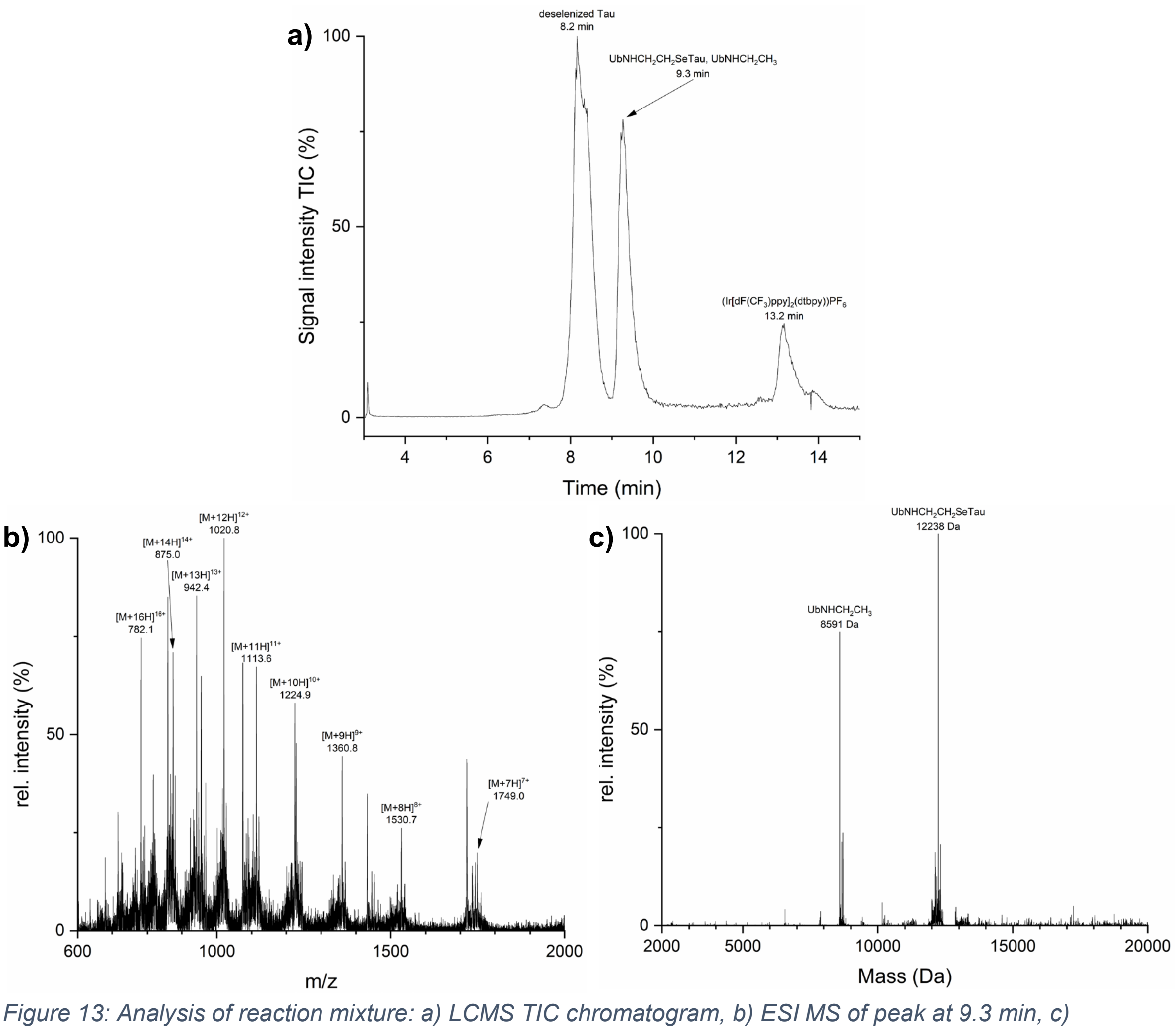

Figure 13: Analysis of reaction mixture: a) LCMS TIC chromatogram, b) ESI MS of peak at 9.3 min, c) Deconvoluted ESI MS of peak at 9.3 min ($UbNHCH_2CH_3$: Exp: 8592 Da, Obs: 8591 Da; $UbNHCH_2CH_2SeTau$: Exp: 12239 Da, Obs: 12238 Da).

containing products were combined and lyophilized. The product (0.97 mg, 79.3 nmol, 49% yield) was recovered as white voluminous solid.

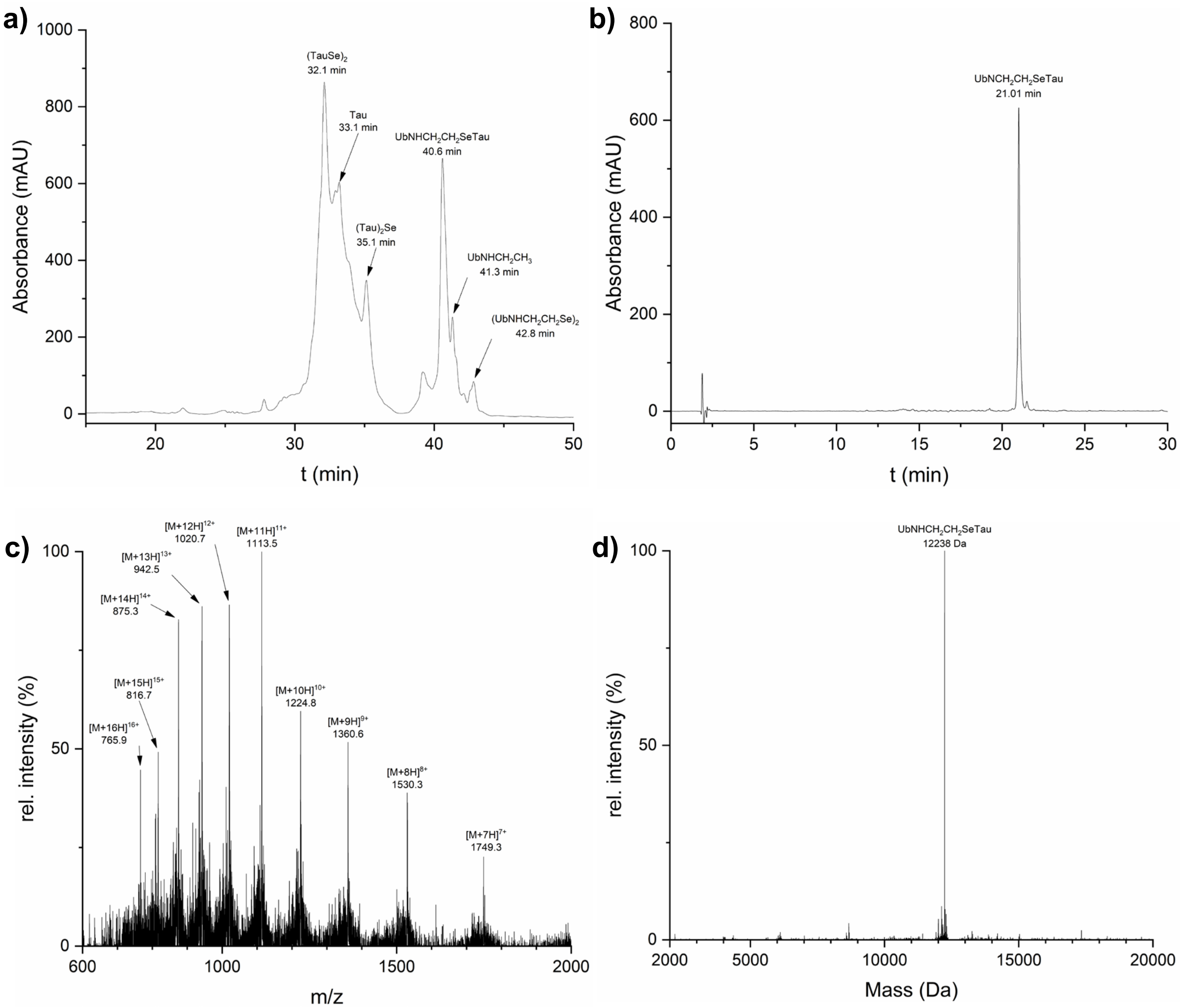

Figure 14: Characterization of UbNHCH$_2$CH$_2$SeTau: a) Preparative chromatogram (Gradient C, 60°C, PerfectSil 300 C4 10 µm, 250x10 mm, 214 nm), b) RP-HPLC at 214 nm, c) ESI MS, d) Deconvoluted ESI MS (UbNHCH$_2$CH$_2$SeTau: Exp: 12239 Da, Obs: 12238 Da).

2.5 Synthesis Ub-Tau heterodiselenides

2.5.1 Synthesis of Ub-SeSe-Tau from Tau-SeS-nitropyridyl and ubiquitin-SeH:

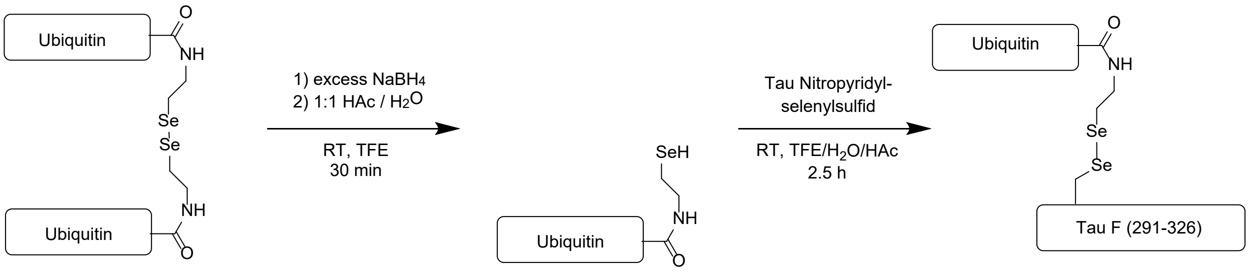

Table 2: Screening of removable reducing agents for the reduction of ($UbNHCH_2CH_2Se)_2$ to yield the corresponding selenol.

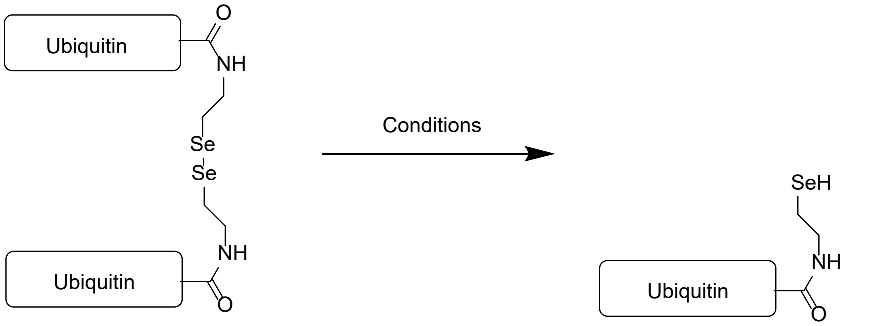

Reagents	Conditions	Result
HCl/Zn	RT, H_2O, 2 h	No reduction
$NaBH_4$	RT, THF, 1 h	No result, low solubility
$NaBH(CF_3COO)_3$	RT, TFA, 6 h	No reduction
$NaBH(CF_3COO)_3$	40°C, TFA, 6 h	Incomplete reduction
**$NaBH_4$**	**RT, TFE, 0.5 h**	**Quantitative reduction**

$(UbNHCH_2CH_2Se)_2$ (0.6 mg, 34.6 nmol) was weighed out and dissolved in 60 µl of trifluoroethanol. Solid $NaBH_4$ (0.6 mg, 15.8 mmol, 460 eq.) was added. A small amount of H_2 gas evolved. After 30 min of reaction at RT on the shaker at 1400 rpm, 100 µl of degassed 1:1 HAc/H_2O was added to quench excess $NaBH_4$. Under flow of Nitrogen, the reaction was allowed to proceed for 90 s. Solid TauSeSnitropyridyl (0.6 mg, 158 nmol,

4.6 eq.) was added, which caused the reaction mixture to become yellow. The reaction vessel was flushed with nitrogen gas and allowed to react for 2.5 h at RT. The reaction

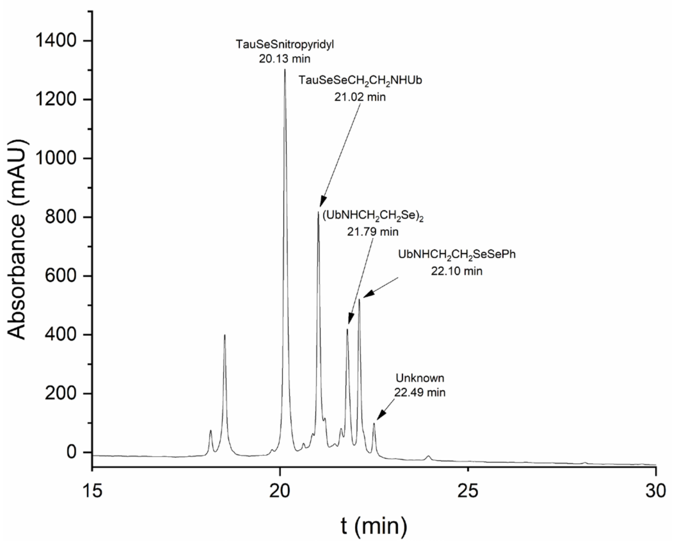

Figure 15: RP-HPLC chromatogram of the crude reaction mixture of UbNHCH2CH2SeH and Tau 5-nitropyridyl selenylsulfide at 214 nm.

mixture was analysed using RP-HPLC and LCMS. LCMS was used to confirm the identity of the species in the RP-HPLC chromatogram. An approximate yield of 50% was calculated by the relative area of all ubiquitin containing HPLC peaks. The side products were identified as $(UbNHCH_2CH_2Se)_2$ and $UbNHCH_2CH_2SeSePh$ (DPDS was introduced as a contaminant of $(UbNHCH_2CH_2Se)_2$) and were caused by air oxidation of the selenol after quenching of the reducing agent.

2.5.2 Photocatalyzed Synthesis of $UbNHCH_2CH_2SeSeTau$ from TauSeSnitropyridyl and $(UbNHCH_2CH_2Se)_2$:

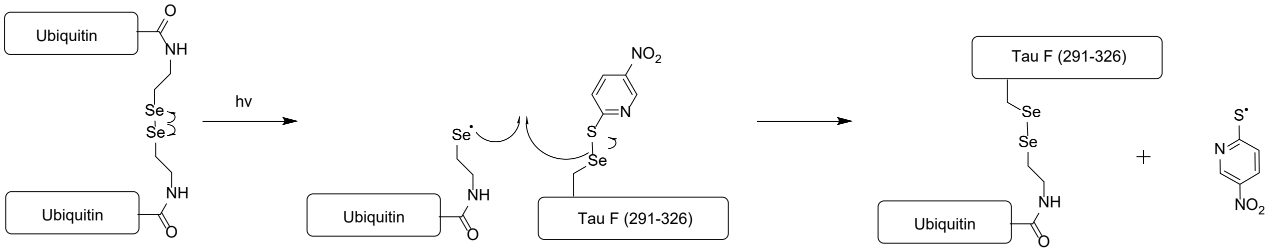

Scheme 2: Proposed mechanism for the photochemical formation of UbNHCH2CH2SeSeTau from TauSeSnitropyridyl and (UbNHCH2CH2Se)2.

0.2 mg of $(UbNHCH_2CH_2Se)_2$ and 0.25 mg TauSeSnitropyridyl were dissolved in 100 µl of water. The reaction mixture was irradiated at 360 nm at an intensity of 166 mW/cm^3 for

20 min. LCMS was measured at 3, 10- and 20 min irradiation. Due to the large amount of side products, this method was deemed unsuccessful.

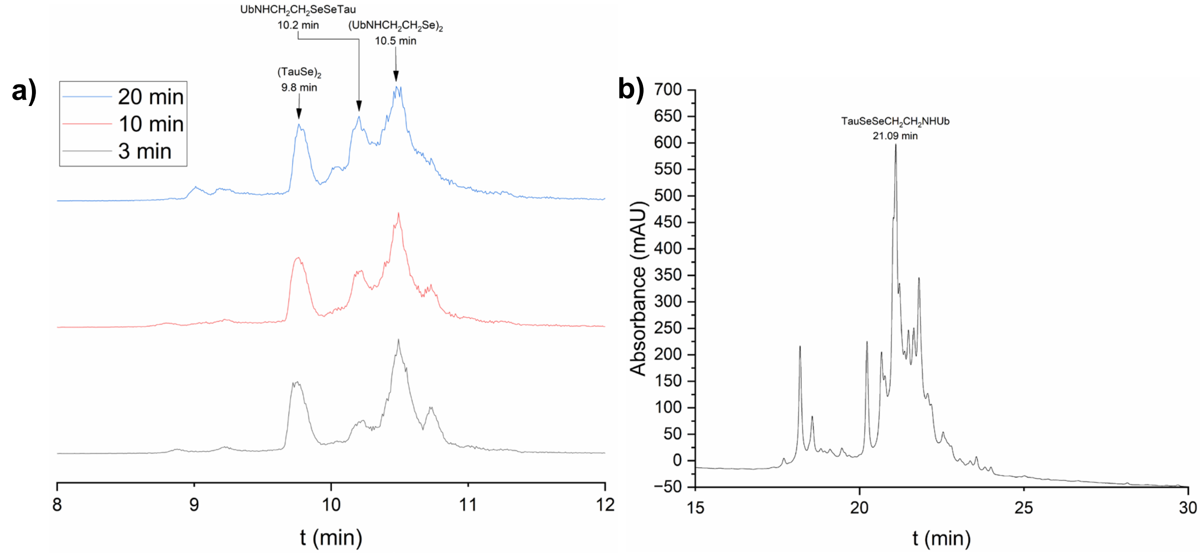

Figure 16: Analysis of the reaction mixture between ($UbNHCH_2CH_2Se)_2$ and TauSeSnitropyridyl after irradiation at 360 nm: a) LCMS TIC chromatogram at 3, 10 ,20 min, b) RP-HPLC chromatogram after 20 min of irradiation at 214 nm.

2.5.3 Synthesis of ubiquitin-Tau heterodiselenide using equilibrium exchange reaction

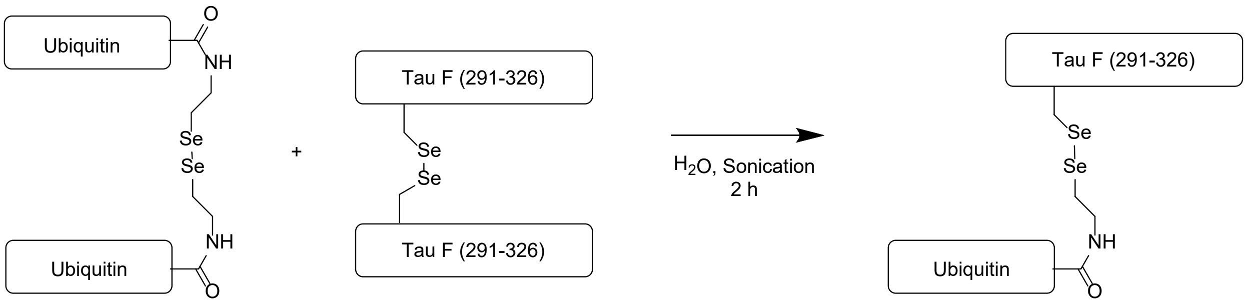

($UbNCH_2CH_2Se)_2$ (10 mg, 0.58 µmol) was weighed out. To this $(TauSe)_2$ (6 mg, 0.82 µmol, 1.43 eq.) were added. The proteins were dissolved in 10 ml of water. The reaction mixture was incubated in the ultrasound bath for 2 h and complete equilibration of the diselenides was confirmed using LCMS and RP-HPLC. The reaction mixture was centrifuged at 5411 × g for 20 min, filtered through a 0.22 µm syringe filter and purified via preparative RP-HPLC (Gradient C, MZ-PerfectSil 300-10-C4, 10×250 mm, 60°C). The product eluted between 43 and 44 min. The fractions were analysed using MS and all

fractions containing starting materials or products were combined and lyophilized. The product (5.3 mg, 0.43 µmol, 37% yield) was recovered as white voluminous solid.

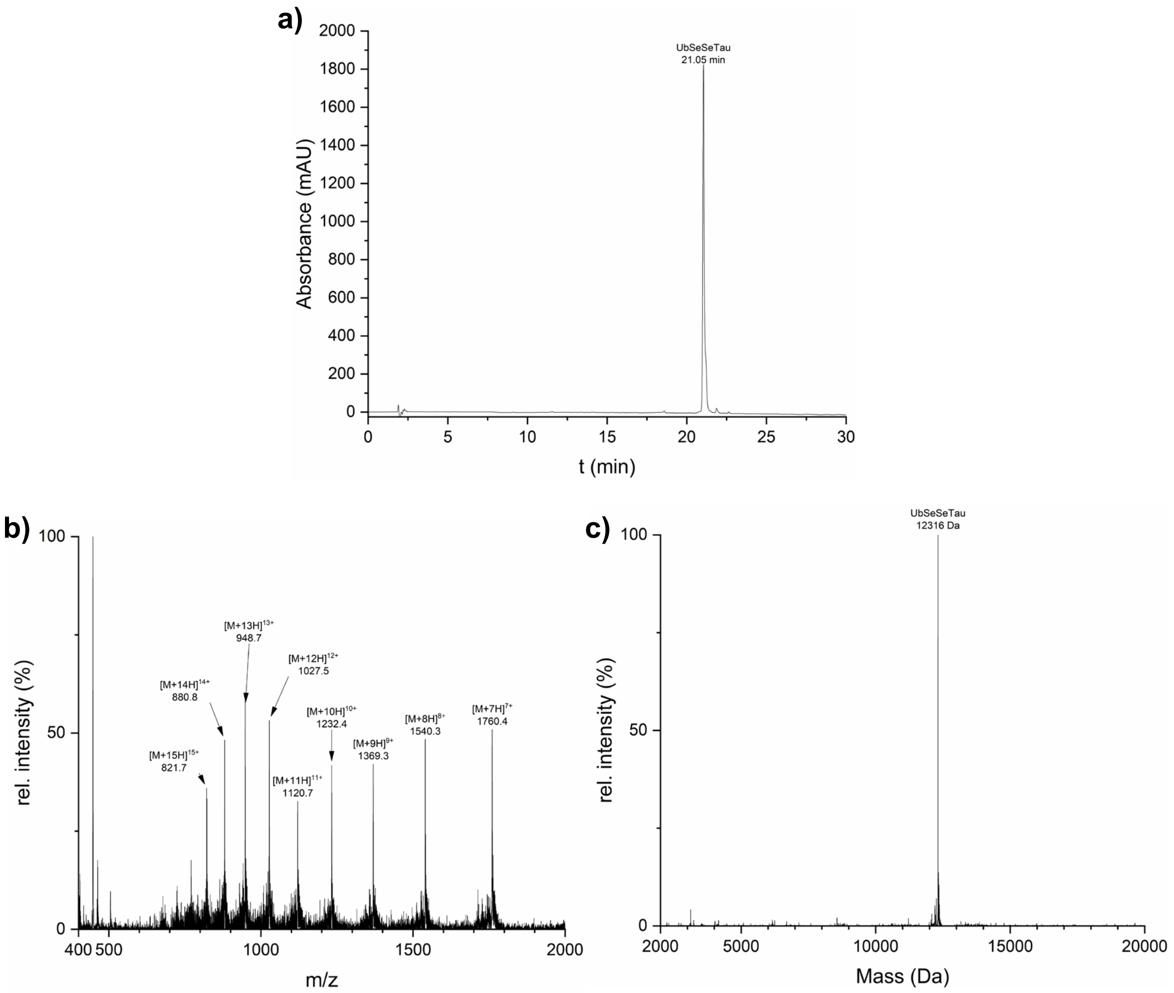

Figure 17: Characterization of UbNHCH2CH2SeSeTau: a) Analytical RP-HPLC chromatogram at 214 nm, b) ESI MS, c) Deconvoluted ESI MS (Exp: 12318 Da, Obs: 12316 Da).

2.6 Synthesis of selenylsulfides

2.6.1 Preparation of Selenylsulfides though exchange of diselenides with thiols

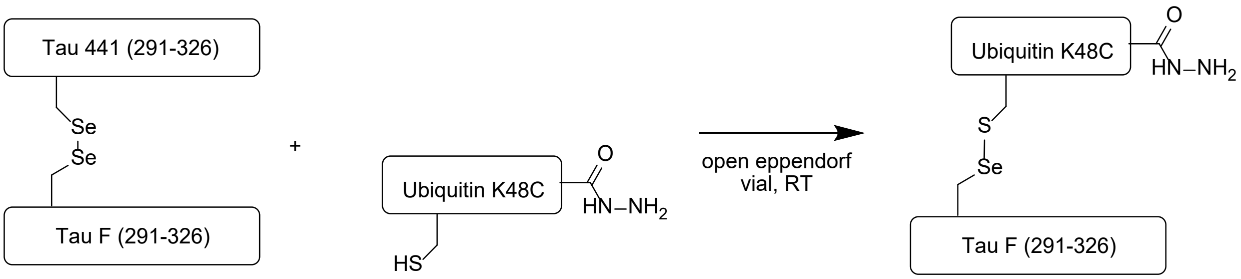

Ubiquitin K48C (1 mg, 0.12 µmol) and $(TauSe)_2$ (3.2 mg, 0.44 µmol, 7.5 eq.) were weighed out and dissolved in 83 µl of 1:1 ACN:H_2O. The reaction was allowed to proceed at RT and progress was checked periodically by LCMS. Once all the solvent was evaporated, more was added. After eight days, less than half of the thiol had been converted to the selenylsulfide. PTA solution in water (5.2 µl, 100 mg/ml, 0.52 mg, 3.3 µmol, 28.3 eq.) as well as $(Ir[dF(CF_3)ppy]_2(dtbpy))PF_6$ solution in ACN (15 µl,

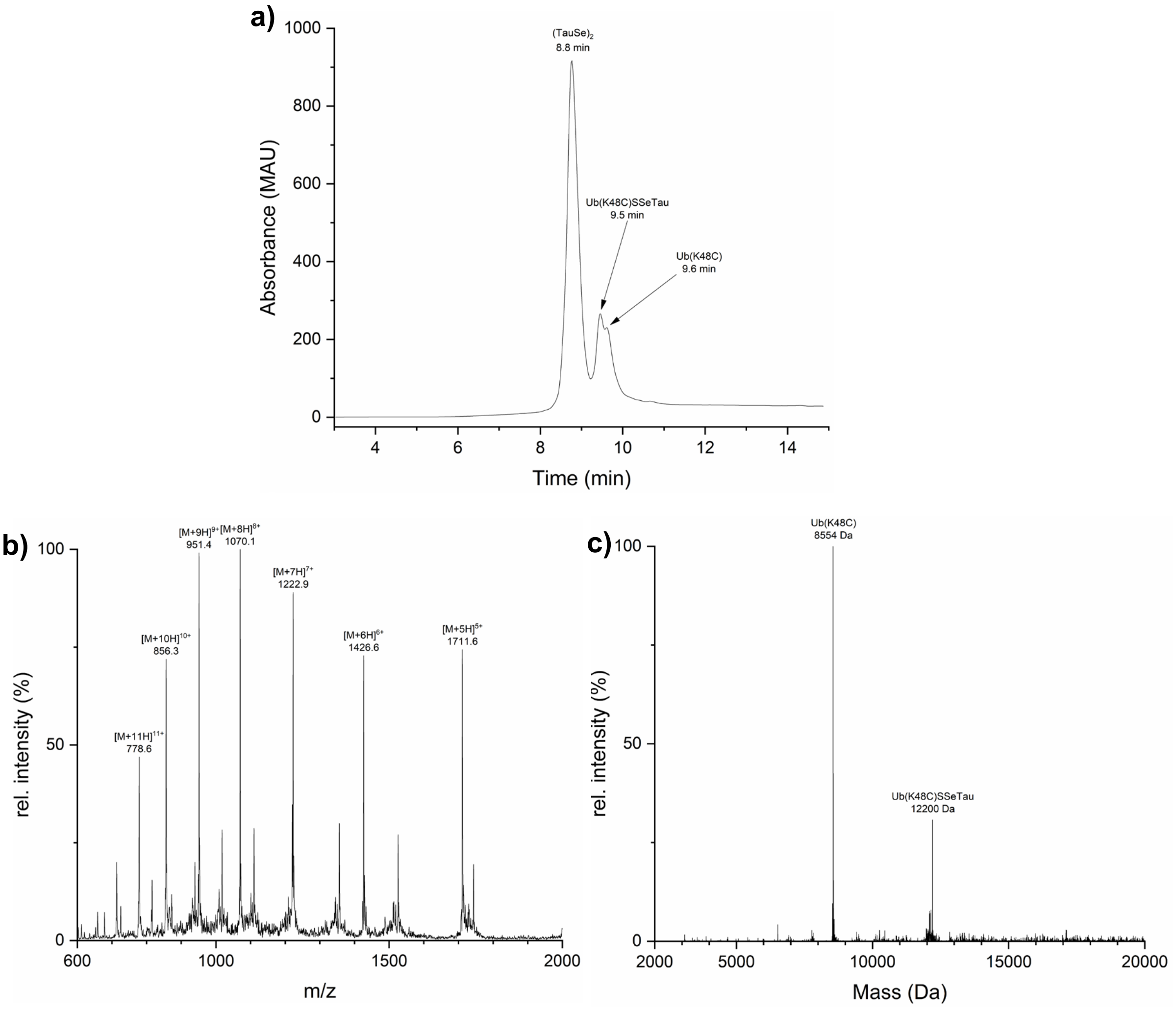

Figure 18: Characterization of an air equilibrated diselenide-thiol exchange reaction using UbK48C and $(TauSe)_2$ after 8 d.: a) LCMS at 214 nm, b) ESI MS of peak at 9.5-9-6 min, c) Deconvoluted ESI MS (Ub(K48C): Exp: 8554 Da, Obs: 8554 Da; Ub(K48C)SSeTau: Exp: 12201 Da, Obs: 12200 Da).

0.5 mg/ml, 7.5 µg, 6.7 nmol, 6 mol%) was added. The mixture was irradiated for 5 min and analysed using LCMS.

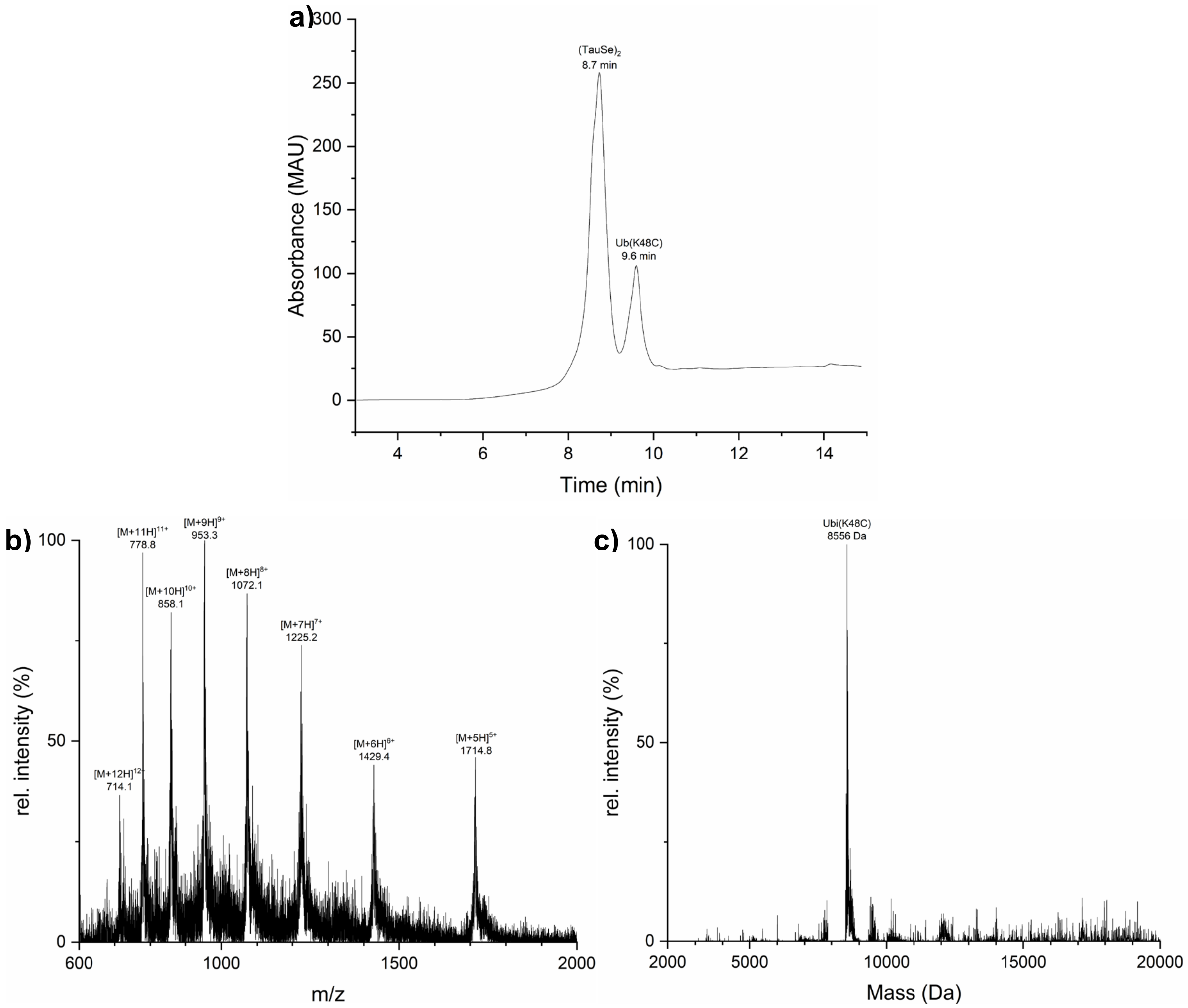

Figure 19: Air equilibrated diselenide-thiol exchange reaction using UbK48C and (TauSe)$_2$ mixture after irradiation: a) LCMS at 214 nm, b) ESI MS of peak at 9.6 min, c) Deconvoluted ESI MS of peak at 9.6

2.6.2 Preparation of selenylsulfides through substitution of 5-nitropyridyl selenylsulfides

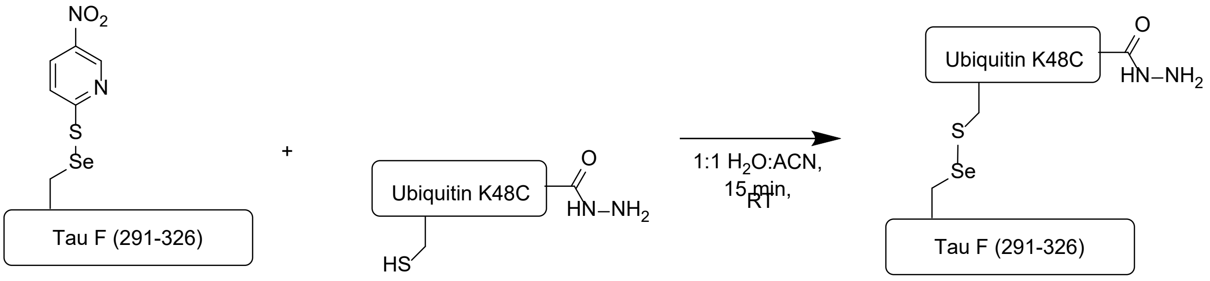

Ub(K48C) (0.75 mg, 87.7 nmol) and TauSeSnitropyridyl (2.4 mg, 0.33 µmol, 3.8 eq.) were weighed out and dissolved in 62 µl of 1:1 ACN:H_2O. The reaction was allowed to proceed for 15 min at RT. LCMS was used to confirm complete conversion to the selenylsulfide. PTA solution (4 µl, 100 mg/ml, 0.4 mg, 2.5 µmol, 29 eq.) was added. (Ir[dF(CF_3)ppy]$_2$(dtbpy))PF_6 solution in ACN (10 µl, 0.5 mg/ml, 5 µg, 4.5 nmol, 5 mol%) was added. The solution was irradiated (5 min, 460 nm).

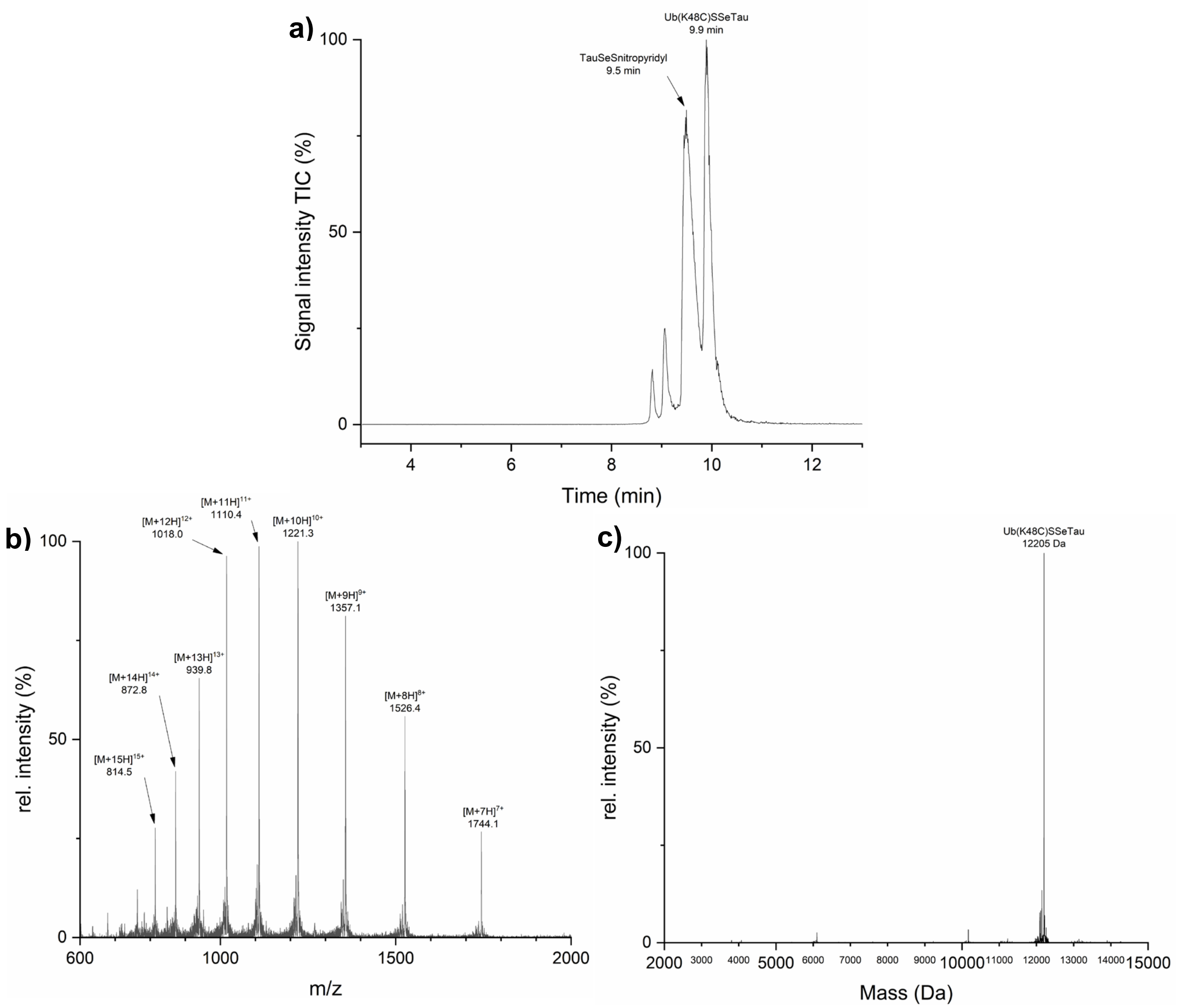

Figure 20: Reaction mixture of Tau 5-nitropyridyl selenylsulfide and ubiquitin K48C after 15 min: a) LCMS TIC chromatogram, b) ESI MS of peak at 9.9 min, c) Deconvoluted ESI MS of peak at 9.9 min (Ub(K48C)SSeTau: Exp: 12201 Da, Obs: 12205 Da).

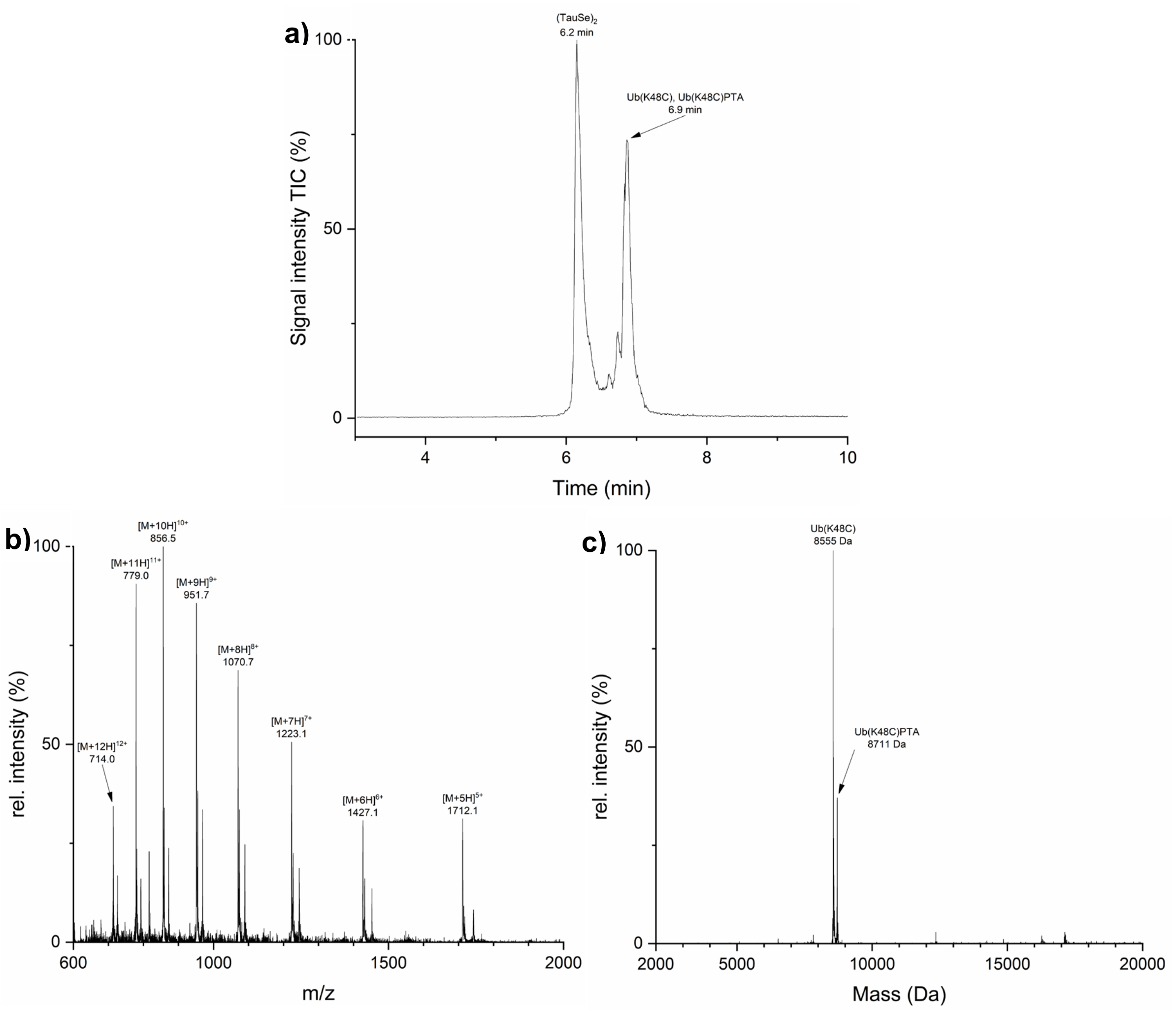

Figure 21: Reaction mixture of Tau 5-nitropyridyl selenylsulfide and ubiquitin K48C after irradiation: a) LCMS TIC chromatogram, b) ESI MS of peak at 6.9 min, c) Deconvoluted ESI MS of peak at 6.9 min (Ub(K48C): Exp: 12201 Da, Obs: 12205 Da; Ub(K48C) PTA: Exp: 8711 Da, Obs: 8711 Da).

2.6.3 Selenalysine linked ubiquitylation as a substrate for DUBs

The USP 21 buffer was composed as follows: 25 mM Tris, 150 mM NaCl, 10 mM DTT, filtered to 0.2 um and frozen at -20°C. The buffer was used to dilute USP21 (196-565) and USP21 (196-565, C221A) from frozen stocks to a concentration of 0.66 µg/µl. The substrates were diluted to a final concentration of 50 µM using water. After 10 min. at RT, the substrate was added to the DUBs solutions to arrive at weight ratio 3:2 of substrate to DUB. After gentle mixing, the solutions were incubated at 37°C for 60 min. Loading buffer is added and the mixtures are briefly heated to 95°C. The samples are loaded onto a NuPAGE™ 4-12% Bis-Tris Protein Gel, 1.5 mm, 15-well (NP0336BOX) and run at a

constant voltage of 200 V for 35 min. The gel was stained using Coomassie blue, washed and imaged (**Figure 22**).

To visualize the cleavage product, Ub-COOH, the experiment was repeated using a 15% acrylamide gel, prepared as in Supplementary Information 1.3 (**Figure 23**). To give more intense bands, the concentration of enzyme and substrate in lanes one and two were increased fourfold.

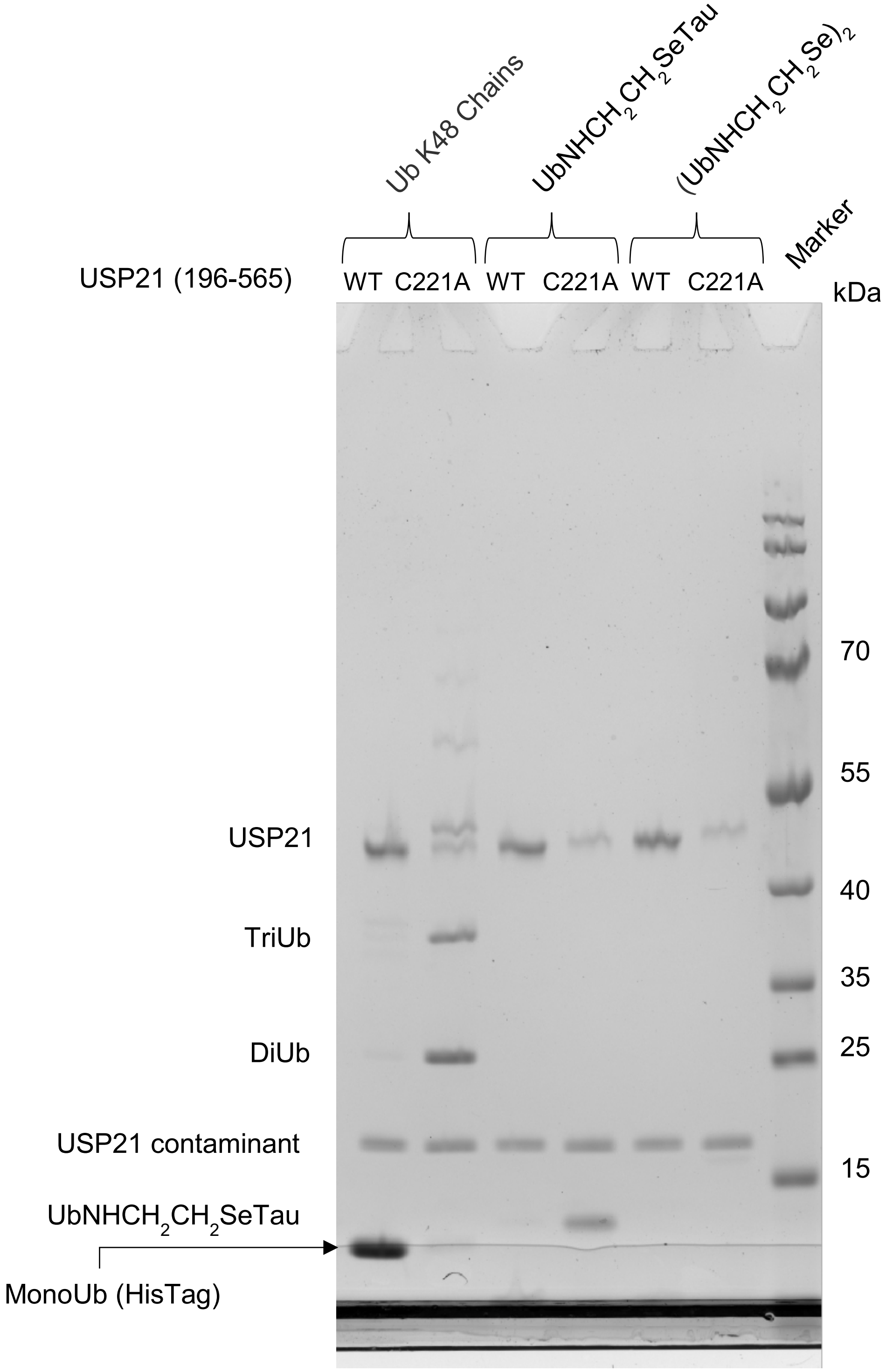

Figure 22: DUB essay: Odd lanes contain the wild type USP21 (196-565), even lanes contain the inactive mutant USP21 (196-565, C221A). The first two lanes (from left to right) were charged with polyubiquitin chains, the following two with selenalysine linked ubiquitylated Tau and the last two with ubiquitin selenocystamine. The very last lane is used for a protein size marker.

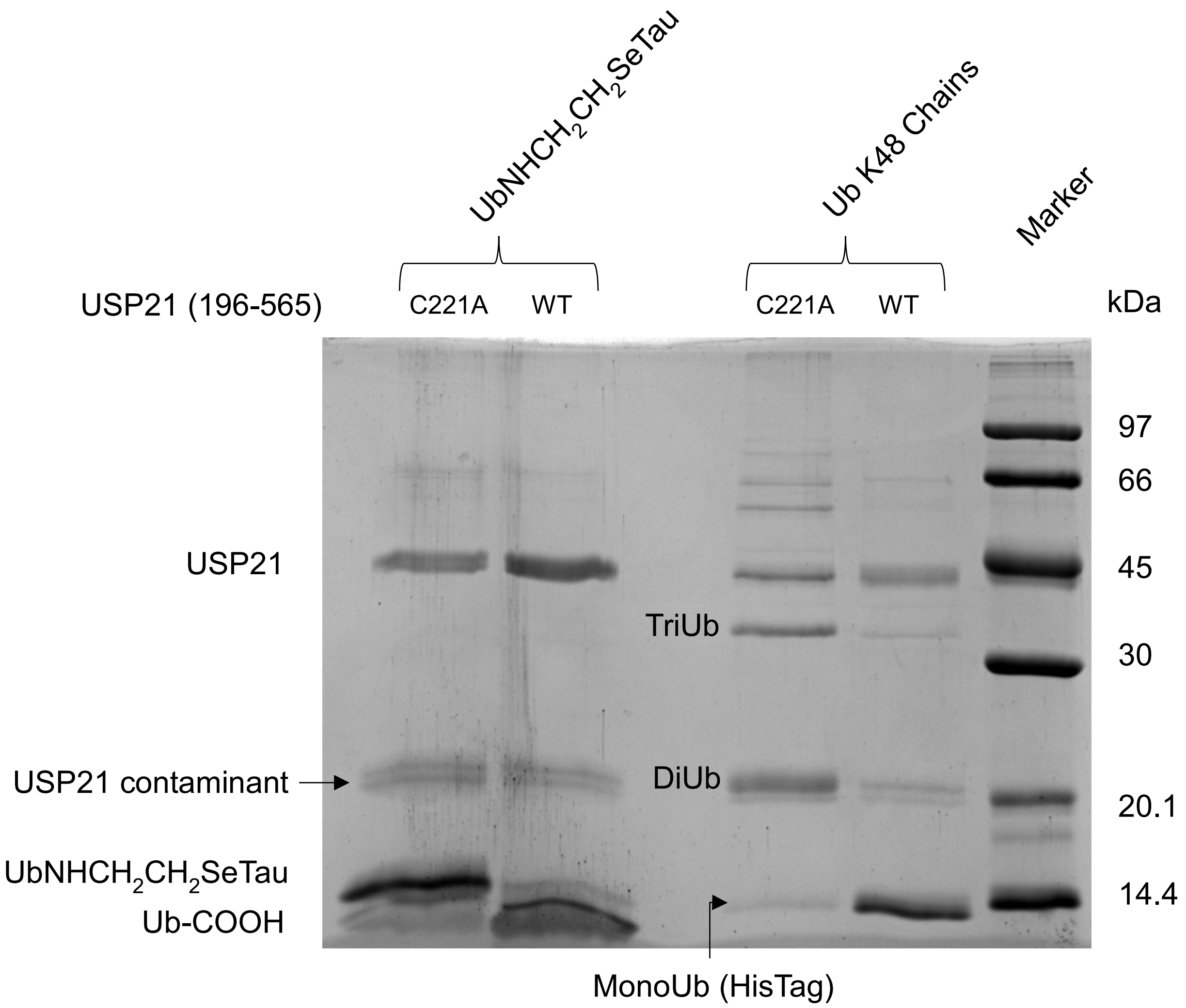

Figure 23: DUB essay using 15% acrylamide gel: Lanes one and four contain the wild type USP21 (196-565), lane three was left empty intentional, lanes two and five contain the inactive mutant USP21 (196-565, C221A). The first two lanes (from left to right) were charged with selenalysine linked ubiquitin, lanes four and five were charged with polyubiquitin chains. The contaminant in lanes one and two, directly above USP21 contaminant band, is likely the homocoupling product ($(UbNHCH_2CH_2)_2Se$).

3 Peptide sequences

3.1 Ub-Mxe-H7-CBD

(H-)MQIFVKTLTGKTITLEVEPSDTIENVKAKIQDKEGIPPDQQRLIFAGKQLEDGRTLSDYNI
QKESTLHLVLRLRGGCITGDALVALPEGESVRIADIVPGARPNSDNAIDLKVLDRHGNPVLADRL
FHSGEHPVYTVRTVEGLRVTGTANHPLLCLVDVAGVPTLLWKLIDEIKPGDYAVIQRSAFSVDC
AGFARGKPEFAPTTYTVGVPGLVRFLEAHHRDPDAQAIADELTDGRFYYAKVASVTDAGVQPV
YSLRVDTADHAFITNGFVSHATGLTGIHHHHHHHSGLNSGLTTNPGVSAWQVNTAYTAGQLVT
YNGKTYKCLQPHTSLAGWEPSNVPALWQLQ(-OH)

(Green = wild type ubiquitin, Red = Mxe intein, Blue = PolyHis tag, Orange = Chitin binding domain)

3.2 Tau F (291-326, K311U)

(H-)GSKDNIKHVPGGGSVQIVYU(Mob)PVDLSKVTSKC(Acm)GSLG(-OH)
